# Measuring information density in interlanguage through entropy analysis

**DOI:** 10.1038/s41598-026-56853-3

**Published:** 2026-06-16

**Authors:** Mohamed Mekheimer

**Affiliations:** Faculty of Education, Beni-Suef University, Beni-Suef, Egypt

**Keywords:** Information density, Entropy, Interlanguage, Second language acquisition, KL divergence, Probabilistic systems, Language and linguistics, Language and linguistics, Mathematics and computing

## Abstract

Interlanguage development is often assessed through structural counts that only partially capture how learner language is organized probabilistically. This study examines whether information-theoretic indicators are sensitive to proficiency-linked distributional differences in argumentative interlanguage writing. It analyzes 200 de-identified argumentative essays: 143 L2 English essays labeled B1, B2, or C1 and 57 genre-matched L1 English essays used as a reference sample. The primary analysis focuses on four operational indicators: lexical entropy (Hlex), grammatical divergence from an L1 reference distribution using POS trigrams (KLgram), compression ratio (CR), and the positional concentration index (PCI). The broader analytical framework also includes an exploratory phraseological layer operationalized as entropy over contiguous 3-word lexical sequences. Descriptive results suggested increasing phraseological dispersion across proficiency levels; however, because more than 90% of these sequences occurred only once within the fixed 250-token windows, the estimates were treated as supplemental and were excluded from the primary inferential analysis. Welch’s ANOVA and Games-Howell post hoc tests indicated ordered group differences in the observed analytic sample. Hlex and PCI increased across proficiency groups, whereas KLgram and CR decreased, suggesting broader lexical dispersion, closer local grammatical alignment with the L1 reference distribution, greater global structural regularity, and stronger early information packaging. The findings are interpreted as evidence of probabilistic reorganization within this bounded argumentative-writing corpus, not as a universal model of L2 development or as a mathematical validation of the measures. They should be read in light of the study’s genre, sampling, annotation, fixed-window, metadata, and data-sharing constraints.

**Supplementary Information:**

The online version contains supplementary material available at 10.1038/s41598-026-56853-3.

## Introduction

 Interlanguage, the developing linguistic system learners construct while moving between prior knowledge and target-language norms, is simultaneously systematic and variable. In second language acquisition (SLA), this development is often described with reference to the Common European Framework of Reference (CEFR), where B1, B2, and C1 denote progressively more advanced proficiency bands. Much SLA work has operationalized development through accuracy, fluency, complexity, lexical richness, vocabulary assessment, and syntactic-complexity indices^1–4^. Yet many commonly used indices count forms, errors, or structural configurations without directly modeling how learner choices are distributed probabilistically. This motivates a complementary lens: information density, understood here as the uncertainty carried by linguistic choices and the way that uncertainty is distributed across lexical, grammatical, and discourse-organizational domains.

Information theory offers formal tools for measuring uncertainty via entropy and for comparing distributions via KL divergence. In linguistic research, entropy-family measures have been used to capture complexity as unpredictability in a probabilistic system, while compression-based approaches approximate Kolmogorov complexity as a holistic measure of information content and redundancy. Juola’s early work introduced compression-based assessment of linguistic complexity, and later work extended this logic to learner-language and corpus-based complexity measurement^5–8^. These tools are especially promising for interlanguage because development can be reframed as a shift in distributions: learners gradually reallocate probability mass toward more contextually appropriate and target-compatible choices, even while different subsystems develop at different rates.

A second motivation comes from research on information distribution in discourse, processing, and L2 pedagogy. Entropy- and surprisal-based measures operationalize information density and information rate by capturing how uncertainty and predictability are managed across unfolding linguistic signals^9–11^. Research on bilingual speech further indicates that information encoding and transmission profiles can differ systematically between L1 and L2 production^12^, and L2 pedagogy research shows that information-density effects may shape discourse processing and comprehension in EFL contexts^13^. Multimodal work also shows that information density is position-sensitive within discourse: density may peak in specific structural locations, such as question beginnings, and may covary with the distribution of visual signals or embodied event encoding^14,15^.

The present study is primarily an applied SLA investigation of interlanguage development approached through a probabilistic lens. It examines whether learner writing from CEFR B1 to C1, compared with genre-matched L1 writing, shows ordered subsystem-specific redistribution of probability mass across lexical, grammatical, structural, and discourse-positional domains in this corpus. Information-theoretic measures are used as diagnostic descriptors for this developmental question, not as objects of mathematical validation in themselves. A secondary methodological aim is to assess whether established measures such as entropy, KL divergence, compression ratio, and PCI are sensitive enough to recover expected proficiency-linked distributional patterns in the present controlled corpus. The manuscript therefore treats the four reported measures as complementary distributional descriptors rather than as a single scalar index of complexity or proficiency.

Several technical terms are used in a narrow analytic sense. Probability mass refers to how often a learner’s available linguistic options are distributed across alternatives. Target-like allocation refers to a learner distribution that more closely resembles the matched L1 reference distribution. Subsystem-specific allocation means that this resemblance may emerge in one layer of language, such as POS-trigram sequencing, but not necessarily in another, such as lexical dispersion. Global structural regularity refers to recurring, compressible organizational patterning across texts. Finally, a bag-of-words representation treats a text as an unordered collection of words and therefore ignores sequential relations among them.

## Research questions and hypothesis

The study was guided by the following research questions:

1. To what extent does lexical information density, operationalized as lexical entropy (Hlex), vary across the B1, B2, C1, and L1 groups?
2. To what extent does grammatical information density, operationalized as grammatical divergence from the native-speaker reference distribution (KLgram), vary across the B1, B2, C1, and L1 groups?
3. To what extent does global structural regularity, operationalized as compression ratio (CR), vary across the B1, B2, C1, and L1 groups?
4. To what extent does discourse-positional information packaging, operationalized as the positional concentration index (PCI), vary across the B1, B2, C1, and L1 groups?
5. Within this corpus, do these four indicators together reveal an ordered proficiency-linked profile from lower-proficiency interlanguage to the L1 reference sample?
6. To what extent can interlanguage writing in this corpus be described as a probabilistic system whose lexical, grammatical, structural, and discourse-positional distributions are reorganized as proficiency increases?

### Hypothesis:

If information-theoretic indicators are sensitive to proficiency-linked distributional reorganization in this corpus, then Hlex and PCI will increase across B1, B2, C1, and L1, while KLgram and CR will decrease across the same ordered groups.

## Literature review

### Entropy, information density, and linguistic complexity

Information-theoretic approaches conceptualize linguistic complexity as the degree of unpredictability in linguistic choices rather than simply the number of structures used. On this view, complexity is a distributional property: a linguistic system is more complex in a given representational space when outcomes are less predictable and more dispersed. However, this does not mean that every developmental gain must produce higher entropy. Different metrics index different facets of organization, including dispersion, sequential dependence, redundancy, and distance from a reference distribution^16–19^.

Compression-based approaches have an older lineage in linguistic complexity research. (Juola^7,8^, introduced compression and related information-theoretic procedures as practical ways of estimating aspects of linguistic complexity when direct calculation of Kolmogorov complexity is impossible. Later work in learner-language research developed this line by using compression to evaluate global regularity and redundancy in SLA production data^20,6,21^.

### KL divergence and proficiency development in L2

A central strand of applied work operationalizes interlanguage development via KL divergence, measuring how far learner distributions deviate from a reference distribution and how this distance changes across proficiency levels. Sun and Wang^22^, propose relative entropy of linguistic complexity as a way to assess L2 proficiency development, arguing that distributional discrimination provides a sensitive index of developmental change beyond traditional complexity metrics. Wang et al.,^21^ extend information-theoretic evaluation by incorporating Kolmogorov complexity metrics alongside entropy-based measures. These studies collectively shift the SLA focus from isolated inventories of forms to how learners distribute probability mass across available options.

The point is not that SLA research historically ignored distribution altogether. Rather, the contrast is methodological: conventional indices in SLA have often relied on counts, ratios, or error-sensitive measures, whereas entropy-family metrics directly estimate the shape of the distribution that generates those counts. This distinction is important because a learner may produce advanced structures sporadically while still relying heavily on a small set of default templates.

### Compression and global regularity in learner language

Compression ratio is used here as a holistic proxy for global structural regularity. A lower compression ratio indicates that the text is more compressible after standardization and therefore contains more recurring structure. This measure should not be interpreted as a direct linguistic construct on its own; rather, it complements entropy and KL divergence by capturing whole-text regularity that may not be visible in feature-by-feature measures^6,8^.

### Sequential dependence and POS-based grammatical modeling

Simple lexical entropy can miss sequential organization because it treats words as distributional outcomes without modeling their local ordering. POS n-grams provide a more sequence-sensitive way to represent short-range grammatical organization while avoiding some of the parser fragility associated with full dependency analysis of learner language. POS-sequence methods are widely used in probabilistic language modeling and information-density research; for example, Demberg et al.,^23^ model syntactic surprisal in conversational production, and von Prince and Demberg^24^, use POS-tag perplexity as a measure of syntactic complexity.

Phraseological organization remains theoretically relevant because multiword patterning can reflect developmental changes in formulaicity and phraseological complexity. However, when contiguous 3-word sequences are extracted from short fixed-length windows without recurrence or dispersion thresholds, the resulting distributions can be dominated by single-occurrence events. The present study therefore retains phraseological entropy only as an exploratory supplemental analysis rather than as a primary inferential metric^25,26^.

### Information density, processing, and modality

Information density is not only a static textual property; it is also related to how language is processed and packaged over time. Jaeger^9^, links syntactic reduction to management of information density, while Levy^11^ explains comprehension difficulty through expectation-based surprisal. Bradlow^12^, shows that L1 and L2 speech differ in information encoding and transmission profiles. Trujillo and Holler^14^, further show that multimodal information density is highest in question beginnings and that early entropy is associated with changes in visual signaling. These findings justify treating discourse position as relevant to information packaging.

Recent EFL listening evidence further supports the view that information density is not only a formal property of linguistic material but also a processing-sensitive feature shaped by discourse organization. Mekheimer^27^, found that an information-rich scientific listening text produced higher comprehension accuracy, faster processing, lower cognitive load, and greater reliance on top-down strategies than a rhetorically complex literary text. Although listening and writing involve different modalities, this evidence reinforces the broader premise that discourse organization can affect how information is packaged, processed, and strategically managed by L2 users.

### Related evidence from mediated language varieties

Entropy measures have also been used to investigate simplification and variation in mediated language, including translated and machine-generated text. Liu et al.,^28^ use entropy to study simplification in translated Chinese. Wang et al.,^29^ apply entropy analysis to lexical and syntactic simplification in translated English. Yao and Fan^30^, compare entropy patterns in ChatGPT, neural machine translation, and human translation across genres. Although these studies are not SLA studies, they demonstrate that entropy-family measures can distinguish varieties, genres, and production conditions through distributional differences.

### Synthesis and gap

Existing work shows that KL divergence can discriminate proficiency groups and model developmental change, that compression approximations can capture holistic redundancy and structure in learner production, and that information density has processing- and discourse-sensitive correlates^12,6,22,14^. What remains underdeveloped is a tightly specified interlanguage account that integrates these measures across lexical, grammatical, structural, and discourse-positional levels while avoiding overclaiming from any single metric.

### Theoretical framework

The theoretical framework conceptualizes interlanguage as a constrained probabilistic system. Learners operate under limits of knowledge, attention, task demands, and processing capacity; consequently, production involves selecting among competing lexical, grammatical, and phraseological options with different probabilities. Development is therefore modeled as redistribution of probability mass rather than as the linear accumulation of discrete rules.

Within this framework, Hlex captures lexical dispersion: higher values indicate a broader and more evenly distributed lexical choice space. KLgram captures grammatical distance from the L1 POS-trigram reference distribution: lower values indicate stronger convergence toward target-like local grammatical sequencing. CR captures global regularity through compressibility: lower values indicate that standardized texts contain more recurring and compressible organization. PCI captures discourse-position packaging by comparing the opening-segment entropy rate with the entropy rate of the full fixed window: higher values indicate a greater concentration of informational load at the beginning of the analytic segment. Exploratory Hphr is treated separately as a descriptive indicator of 3-word lexical-sequence dispersion rather than as a primary inferential metric. These directional interpretations explain why some indicators are expected to rise with proficiency and others to fall.

The framework therefore predicts subsystem-specific reorganization. Lexical entropy may increase as the learner repertoire expands, while grammatical divergence and compression ratio may decrease as production becomes more constrained, conventionalized, and globally organized. PCI may increase when writers learn to front-load thesis, stance, and topic-setting information in argumentative writing. The framework does not treat “more entropy” as inherently better; each metric has meaning only within its representational space and reference relation.

## Methods

### Research design

This study employs a quantitative, corpus-based design to operationalize information density in interlanguage using entropy-family measures, KL divergence, compression ratio, and positional concentration. The central assumption is that interlanguage reflects constrained probabilistic choice. The study therefore does not primarily count structures or errors; instead, it measures how distributions are organized in a controlled argumentative-writing corpus and how those distributions differ across proficiency-labeled groups.

### Participants and corpora

The corpus materials comprised a restricted, de-identified L2 source corpus of 150 argumentative essays produced by B1, B2, and C1 learners, together with a genre-matched L1 reference corpus. The inferential dataset used in the manuscript contains the observed analytic sample after quality-control screening, fixed-window extraction, and final analytic proficiency coding. This analytic sample includes 200 essays: 143 L2 English essays and 57 L1 English essays. The inferential groups are B1 (*n* = 45), B2 (*n* = 55), C1 (*n* = 43), and L1 (*n* = 57). Thus, the internal corpus record documents the source learner texts, whereas the reported statistical files document the empirical analytic grouping used for all inferential tests. The L1 reference corpus serves as a genre- and task-matched benchmark, not as an exhaustive native norm. Public supplementary materials are limited to shareable derived numeric variables, aggregate descriptive and inferential outputs, preprocessing notes, and robustness documentation. They do not include participant-generated essays, text excerpts, or any files covered by consent restrictions.

The accompanying metadata are not sufficiently comprehensive to support strong developmental generalization beyond the sampled texts. Although the essays are classified by CEFR level, the corpus does not preserve full documentation of the original proficiency-assignment procedures, prompt-level identifiers, or participant-level background variables for all cases. CEFR grouping is therefore treated as a corpus-encoded proficiency label rather than as a newly validated measurement instrument. This constraint is important for interpreting group differences as corpus-based evidence of proficiency-linked distributional patterning rather than as a population-level developmental trajectory.

### Data preparation and linguistic annotation

All texts underwent a uniform processing pipeline. Texts were tokenized, sentence-segmented, lemmatized, and POS-tagged with spaCy (v3.7.2) using the en_core_web_md model. A stratified manual audit of 10% of the essays per group was used to check the plausibility of automated annotation. Crucially, lemmatization and POS tagging were treated as analytical labeling, not as learner-language correction. The NLP pipeline categorized and lemmatized tokens as they appeared in the learner texts; it did not rewrite sentences into target-like English, regularize non-canonical syntax, or reconstruct intended native-speaker forms before analysis. Consequently, the probability distributions reflect the learner’s actualized interlanguage system, including error-bearing and non-canonical productions, rather than an idealized corrected version. Conservative normalization was restricted to technical preprocessing anomalies, such as tokenization artifacts, spacing irregularities, and encoding noise.

### Units of analysis

Primary information density was operationalized through four reported indicators, while phraseological information density was retained as an exploratory supplemental analysis. This distinction preserves the theoretical relevance of multiword organization without allowing sparse phraseological estimates to drive the article’s primary claims.

1. Lexical layer (Hlex). Distributions were computed over lemmas to model repertoire size and dispersion of lexical choice.
2. Grammatical layer (KLgram). Distributions were computed over POS trigrams drawn from the Universal POS tagset to capture short-range morphosyntactic sequencing. POS trigrams were preferred over POS bigrams because they preserve one additional step of local context, and they were preferred over dependency relations because dependency parses are more vulnerable to annotation error in non-canonical learner sentences. This choice is consistent with POS-sequence approaches in information-density and syntactic-complexity research^23,24^.
3. Phraseological layer (Hphr; exploratory). To preserve the theoretical breadth of the framework while avoiding overinterpretation, phraseological information density was examined as an exploratory supplemental analysis. The measure was operationalized as Shannon entropy over contiguous 3-word lexical sequences extracted from each fixed 250-token analysis window. Unlike the grammatical layer, which relies on abstract POS trigrams, this exploratory layer captures surface-level multiword sequencing in the produced texts. Because the analysis did not impose corpus-wide recurrence or dispersion thresholds, these units are described as 3-word lexical sequences rather than recurrent lexical bundles in the stricter corpus-linguistic sense.
4. Global structural layer (CR). Standardized text strings were compressed with LZMA, and the compression ratio was interpreted as a proxy for whole-text regularity and redundancy.
5. Discourse-position layer (PCI). To control the sensitivity of entropy estimates to segment length, all primary analyses used a fixed-length static window design. For each essay, one contiguous segment of exactly 250 tokens was extracted from the center of the text. This window was neither moving/sliding nor random: the same central-extraction rule was applied across groups, and each token in the analytic segment was counted once. Within this fixed window, PCI operationalized information front-loading by comparing an Opening Segment (the first 50 tokens) with the Global Window (all 250 tokens). PCI was defined as the ratio of entropy rates, [H(opening)/50] divided by [H(global)/250]. Values near 1.00 indicate broadly even information density, values below 1.00 indicate a relatively diluted or redundant opening, and values above 1.00 indicate that informational load is front-loaded in the opening segment.

### Information-theoretic measures

Shannon entropy (H) was calculated to quantify dispersion. Higher entropy reflects a more even distribution across outcomes, whereas lower entropy indicates concentration around fewer options. KL divergence was used to quantify directed grammatical distance between learner POS-trigram distributions and the L1 reference distribution. Additive smoothing was applied because KL divergence is undefined when the reference distribution assigns zero probability to learner-observed events. Compression ratio was used as a holistic proxy for redundancy and global structural regularity. PCI was computed as a ratio of entropy rates rather than as a ratio of raw entropies: because the opening segment contains 50 tokens and the global window contains 250 tokens, entropy was normalized by segment length before comparison.

This rate-based formulation allows PCI to index the relative density of information in the opening of the fixed window without confounding the interpretation with unequal segment sizes. For the exploratory phraseological analysis, Shannon entropy was also computed over contiguous 3-word lexical sequences; however, phraseological KL divergence was not modeled because the sequence distributions were too sparse and zero-cell sensitivity would make divergence estimates excessively dependent on smoothing choices. The inferential framework therefore consisted of Hlex, KLgram, CR, and PCI as primary indicators, with Hphr retained only as a descriptive phraseological indicator.

### Statistical analysis

Group-level differences were tested across the observed analytic sample reported above. Because Shapiro-Wilk and Levene diagnostics indicated departures from normality and homogeneity for several variables, the primary inference used Welch’s ANOVA followed by Games-Howell pairwise comparisons. Effect sizes are reported as eta-squared values derived from the omnibus group partitions, alongside the robust Welch tests used for inference. These effect sizes describe the magnitude of group separation within the analytic sample and are not interpreted as population-invariant estimates. This reporting strategy is consistent with recommendations to interpret information-theoretic measures through distribution-sensitive statistical summaries rather than through a single scalar estimate^31^. Games-Howell tables report mean differences, standard errors, approximate t statistics, Welch-Satterthwaite degrees of freedom, and adjusted p values.

### Reliability, robustness, and sensitivity checks

Several checks strengthened measurement credibility. Entropy was recomputed under token and lemma representations. Key descriptive patterns were compared under POS bigrams and trigrams. KL divergence was examined under different smoothing parameters. For compression metrics, preprocessing was standardized so that differences reflected linguistic patterning rather than formatting artifacts. Interpretation was anchored in converging evidence across indicators because entropy may increase under both productive diversification and noisy instability.

### Ethical considerations

The study protocol was reviewed and approved by the Institutional Review Board of the Faculty of Education, Beni-Suef University (Approval No. BSU-FoE-004-01-01-2016). All methods were carried out in accordance with relevant guidelines and regulations. Informed consent was obtained from all participants and/or their legal guardians prior to data collection and analysis. All learner data were anonymized prior to analysis. Participant-generated essays were treated as restricted research data because the consent conditions did not authorize unrestricted public release of the texts. The study therefore reports only aggregated findings and shareable derived outputs and does not disclose identifying personal, contextual, or text-level materials.

## Results

### Descriptive profile

Table 1 reports the descriptive profile for the observed analytic sample across the four primary indicators. Within this sample, the reported indicators show an ordered pattern from B1 to L1. Hlex increases across proficiency levels, indicating broader lexical dispersion. KLgram and CR decline across the same ordered groups, indicating closer approximation to the L1 reference distribution and greater global structural regularity. PCI also rises across levels, indicating that the entropy rate of the opening segment becomes higher relative to the entropy rate of the full fixed window.

**Table 1:** Descriptive statistics by group (Mean, SD).

Group	Hlex	KLgram	CR	PCI
B1 (*n* = 45)	4.131 (0.087)	1.852 (0.089)	0.662 (0.016)	0.894 (0.032)
B2 (*n* = 55)	4.404 (0.113)	1.523 (0.132)	0.620 (0.020)	1.044 (0.058)
C1 (*n* = 43)	4.837 (0.035)	0.966 (0.030)	0.579 (0.009)	1.191 (0.017)
L1 (*n* = 57)	5.202 (0.148)	0.226 (0.281)	0.526 (0.022)	1.268 (0.035)

Note. Hlex = lexical entropy; KLgram = grammatical KL divergence from the L1 POS-trigram reference distribution; CR = compression ratio; PCI = positional concentration index.

#### Exploratory supplemental analysis of phraseological entropy

Table 2 reports the exploratory descriptive profile for phraseological entropy over contiguous 3-word lexical sequences within the fixed 250-token analysis windows. The descriptive pattern shows a monotonic increase in Hphr from B1 to L1, rising from 3.21 in the B1 group to 4.32 in the L1 group. At a descriptive level, this pattern is compatible with broader multiword-sequence dispersion at higher proficiency. However, the accompanying distributional diagnostics caution against treating this trend as a primary inferential result. Sequence type/token ratios were extremely high, ranging from 0.88 to 0.98, and more than 90% of observed sequences were hapax items in every group. These values indicate that the probability distributions were dominated by single-occurrence events rather than by recurrent phraseological patterns.

Accordingly, the phraseological layer is interpreted only as a secondary descriptive indicator of sequence dispersion. It was not retained in the primary inferential framework because sparse high-dimensional distributions can produce unstable entropy estimates when the number of possible events is large relative to the number of observations^32^. This sparsity problem is especially consequential for divergence-based modeling: many 3-word sequences observed in learner texts may be absent from the L1 reference distribution, creating zero-probability events and making phraseological KL divergence estimates excessively dependent on smoothing choices.

**Table 2:** Exploratory descriptive statistics for 3-word lexical-sequence entropy.

Group	*n*	Mean Hphr	SD	Sequence Type/Token Ratio	Hapax Sequences (%)
B1	45	3.21	0.42	0.88	91.2
B2	55	3.56	0.38	0.92	94.5
C1	43	3.98	0.25	0.96	97.8
L1	57	4.32	0.31	0.98	98.2

Note. Hphr = Shannon entropy over contiguous 3-word lexical sequences. Sequence type/token ratio indexes diversity among observed 3-word sequences; hapax sequences are sequences occurring once within the analysis window.

### Assumption checks

Before testing omnibus group differences, the homogeneity-of-variance assumption was examined for each of the four primary information-density indicators. As shown in Table 3, Levene’s tests were significant for Hlex, KLgram, CR, and PCI, indicating unequal variances across proficiency groups. This diagnostic result justified the use of Welch’s ANOVA and Games–Howell post hoc comparisons as the primary inferential procedures, because these tests are more appropriate when the equal-variance assumption is violated.

**Table 3:** Homogeneity of variance across groups.

Metric	Levene F	*p*
Hlex	6.67	< 0.001
KLgram	14.23	< 0.001
CR	3.31	0.021
PCI	4.14	0.007

Note. Because variance homogeneity was violated for all four focal metrics, Welch’s ANOVA and Games-Howell post hoc tests were retained as the primary inferential procedures.

### Omnibus group effects

See Table 4.

**Table 4:** Omnibus tests of group differences.

Metric	Test	F (eta-squared)	df	*p*
Hlex	Welch ANOVA	1181.83 (0.937)	3, 98.92	< 0.001
KLgram	Welch ANOVA	1679.02 (0.933)	3, 94.28	< 0.001
CR	Welch ANOVA	503.30 (0.889)	3, 104.61	< 0.001
PCI	Welch ANOVA	1323.29 (0.929)	3, 104.75	< 0.001

Note. The large effect sizes indicate that proficiency-group membership accounts for a substantial portion of variance in each information-density indicator within this corpus. These effects describe the observed analytic sample and should not be interpreted as population-level estimates without replication.

### Games-howell post hoc tests for hlex

See Table 5.

**Table 5:** Games-howell pairwise comparisons for hlex.

Comparison	Mean diff	SE	t	df	*p*
B1-B2	-0.273	0.020	-13.64	97.67	< 0.001
B1-C1	-0.706	0.014	-50.34	58.41	< 0.001
B1-L1	-1.071	0.024	-45.56	93.06	< 0.001
B2-C1	-0.433	0.016	-26.82	66.77	< 0.001
B2-L1	-0.798	0.025	-32.14	104.54	< 0.001
C1-L1	-0.365	0.020	-17.97	64.14	< 0.001

Note. Mean diff = mean difference (G1-G2). Games-Howell pairwise comparisons report approximate t statistics, Welch-Satterthwaite degrees of freedom, and adjusted p values.

### Games-howell post hoc tests for KLgram

See Table 6.

**Table 6:** Games-howell pairwise comparisons for KLgram.

Comparison	Mean diff	SE	t	df	*p*
B1-B2	0.329	0.022	14.82	94.77	< 0.001
B1-C1	0.886	0.014	63.13	54.28	< 0.001
B1-L1	1.626	0.040	41.15	69.70	< 0.001
B2-C1	0.557	0.018	30.31	61.03	< 0.001
B2-L1	1.297	0.041	31.44	80.19	< 0.001
C1-L1	0.740	0.037	19.73	57.69	< 0.001

Note. Mean diff = mean difference (G1-G2). Games-Howell pairwise comparisons report approximate t statistics, Welch-Satterthwaite degrees of freedom, and adjusted p values.

### Games-howell post hoc tests for CR

See Table 7.

**Table 7:** Games-howell pairwise comparisons for CR.

Comparison	Mean diff	SE	t	df	*p*
B1-B2	0.042	0.004	11.67	97.96	< 0.001
B1-C1	0.083	0.003	30.16	69.93	< 0.001
B1-L1	0.136	0.004	36.12	99.39	< 0.001
B2-C1	0.041	0.003	13.55	78.80	< 0.001
B2-L1	0.094	0.004	23.68	109.62	< 0.001
C1-L1	0.053	0.003	16.45	78.45	< 0.001

Note. Mean diff = mean difference (G1-G2). Games-Howell pairwise comparisons report approximate t statistics, Welch-Satterthwaite degrees of freedom, and adjusted p values.

### Games-howell post hoc tests for PCI

See Table 8.

**Table 8:** Games-howell pairwise comparisons for PCI.

Comparison	Mean diff	SE	t	df	*p*
B1-B2	-0.150	0.009	-16.37	86.89	< 0.001
B1-C1	-0.297	0.005	-54.70	67.65	< 0.001
B1-L1	-0.374	0.007	-56.23	97.81	< 0.001
B2-C1	-0.147	0.008	-17.84	65.50	< 0.001
B2-L1	-0.224	0.009	-24.64	88.12	< 0.001
C1-L1	-0.077	0.005	-14.50	85.37	< 0.001

Note. Mean diff = mean difference (G1-G2). Games-Howell pairwise comparisons report approximate t statistics, Welch-Satterthwaite degrees of freedom, and adjusted p values.

### Summary of developmental reorganization

Across the four focal indicators, the results show a consistent ordered profile within this corpus. Hlex rises, KLgram falls, CR falls, and PCI rises. This combination suggests that learner language in the observed argumentative-writing sample becomes more lexically expansive while also becoming more grammatically aligned with the L1 reference distribution, more globally regular, and more strongly organized across discourse position. The overall pattern is best described as coordinated probabilistic reorganization in this corpus rather than as a universal scalar increase in complexity.

## Discussion

The present study investigated whether information-theoretic indicators can capture expected proficiency-linked distributional differences in a controlled argumentative-writing corpus. The results support this narrower and more testable claim within the observed analytic sample. Hlex, KLgram, CR, and PCI each exhibited systematic and statistically robust variation across proficiency levels. Crucially, the developmental profile was not reducible to a single monotonic increase in complexity; it was more accurately characterized as coordinated reorganization across multiple representational domains.

At the lexical level, entropy increased significantly across proficiency levels, indicating that writers in the higher-proficiency groups distributed probability mass across a broader range of lexical choices. This pattern aligns with information-theoretic accounts in which entropy indexes dispersion across outcomes^5^ and with SLA accounts that view lexical development as progressive expansion and redistribution of the learner’s choice space. The remaining C1-L1 gap suggests that advanced learner writing in this corpus still differs from the L1 benchmark in the balance of lexical distributions.

In contrast, KLgram decreased sharply as proficiency increased. This finding is consistent with the hypothesis that grammatical development involves convergence toward target-like local sequencing. Using KL divergence, the study measured directed distance between learner POS-trigram distributions and the L1 reference distribution. The significant downward trend is compatible with Sun and Wang’s^22^ claim that relative entropy can serve as a sensitive indicator of L2 proficiency development. The coexistence of rising lexical entropy and falling grammatical divergence is theoretically important because it shows that interlanguage does not become uniformly more variable or uniformly more constrained.

The findings for CR are also consistent with the reorganization account. Compression ratio declined across proficiency levels, indicating that learner texts in the higher-proficiency groups were more compressible and globally regular after standardization. This pattern aligns with earlier work showing that compression-based measures can capture holistic structure and redundancy in learner language^20,6,8,21^. The decrease in CR does not contradict the increase in Hlex; rather, it suggests that lexical expansion can coexist with greater whole-text regularity in the observed corpus.

The PCI findings extend the analysis to discourse-position packaging. Because PCI is a ratio of entropy rates, it identifies whether the opening of the fixed analytic window is informationally denser or more diluted than the window as a whole. The increase from B1 (M = 0.894) to L1 (M = 1.268) indicates a shift from relatively low-density openings to stronger information front-loading in the observed sample. At lower proficiency, opening segments may rely more heavily on predictable, formulaic, or prompt-recycling material, which yields a PCI below 1.00. At higher proficiency, writers in this corpus appear more able to establish stance, frame the topic, and introduce lexically and grammatically informative material early in the analytic span, producing a PCI above 1.00. This interpretation is consistent with work showing that information density is position-sensitive in discourse and connected to processing demands^9,11,14^.

It also aligns with recent EFL listening evidence showing that information-rich discourse can reduce perceived processing effort and support more global, top-down comprehension strategies, whereas rhetorically complex discourse increases repair-oriented processing demands^27^.

The study’s central contribution is therefore developmental and applied: it examines whether interlanguage writing in this corpus exhibits systematic probabilistic reorganization across proficiency levels. The results are consistent with that claim by showing coordinated change in lexical dispersion, grammatical alignment, structural regularity, and discourse-position packaging. A secondary contribution is methodological. The findings provide evidence that established information-theoretic measures can be diagnostically useful for identifying proficiency-linked distributional differences in this controlled argumentative-writing corpus. This should be understood as evidence of applied diagnostic utility for the present SLA problem, not as mathematical validation of entropy, KL divergence, or compression theory as such.

### Limitations

Several limitations should be acknowledged. First, the corpus is restricted to argumentative writing, so the reported distributions should not be generalized automatically to other genres, modalities, task ecologies, or instructional settings. Second, the source L2 corpus and the empirical analytic dataset should be distinguished: the internal corpus record preserves the source-text inventory, whereas the inferential analyses use the observed analytic grouping reported in the statistical files after screening, fixed-window extraction, and analytic proficiency coding. Participant-generated essays and text-level files are not included in the public supplementary materials because the original consent conditions did not authorize unrestricted public release. Third, the archived metadata are not sufficiently rich for all cases; learners’ L1 backgrounds, age of acquisition, context of L2 use, writing histories, and fully recoverable prompt identifiers were not available for modeling. Fourth, although the phraseological layer was retained as an exploratory descriptive component, it was not incorporated into the primary inferential framework. The fixed 250-token windows yielded highly sparse 3-word sequence distributions, with over 90% of observed sequences occurring only once. This pattern limited the stability of entropy estimates and made divergence-based reference modeling insufficiently robust for confirmatory interpretation. Fifth, the L1 reference corpus is a matched benchmark rather than a universally representative native norm.

Additional constraints concern measurement and inference. Although the annotation procedures were suitable for large-scale analysis, non-canonical learner language, especially at lower proficiency levels, may still introduce tagging noise that affects entropy and divergence estimates. The fixed-window approach improved comparability across texts but may have reduced the visibility of discourse phenomena that emerge only in longer spans or across full essays. The cross-sectional corpus design cannot establish within-learner developmental trajectories over time, and the CEFR labels are treated as corpus-encoded proficiency categories rather than as independently revalidated measurements. Finally, because consent-restricted texts cannot be openly released, reproducibility depends on the public availability of derived numeric variables, aggregate statistical outputs, scripts, and transparent preprocessing documentation rather than on unrestricted access to the original learner essays.

### Implications

The findings have theoretical, methodological, and pedagogical implications, provided they are interpreted within the limits of the corpus. Theoretically, they support a reconceptualization of interlanguage as a probabilistic, multi-layered system rather than a collection of isolated forms or errors. Methodologically, the study shows that entropy-family measures can be combined to describe dispersion, grammatical distance, compressibility, and discourse packaging within one analytic framework. Pedagogically, the results suggest that learner progress should not be reduced to a single proficiency score or a narrow notion of complexity; learners may advance differently across lexical dispersion, grammatical convergence, structural regularity, and discourse packaging. These implications remain provisional and should be tested in larger, longitudinal, genre-diverse, and metadata-rich corpora.

## Conclusion

The present study offers an empirically grounded account of interlanguage development as probabilistic reorganization within a bounded argumentative-writing corpus. Its primary contribution is an applied SLA account of how learner writing differs across proficiency levels when development is viewed distributionally. Across the four reported subsystems, lexical entropy increased as the range of lexical choices broadened; grammatical KL divergence decreased as sequential patterns moved closer to the L1 reference distribution; compression ratio declined as texts became more structurally regular and compressible; and PCI increased as the opening segment carried a higher entropy rate relative to the full fixed window.

Read alongside prior work on relative entropy, compression-based complexity, and multidimensional complexity modeling, this pattern supports the view that development can be understood as coordinated distributional change rather than as isolated gain in a single domain^2,22,21^. The manuscript also demonstrates the diagnostic utility of information-theoretic measures for capturing proficiency-linked interlanguage organization in the present corpus. At the same time, these findings do not justify a universal scalar notion of complexity, a population-wide model of L2 development, or unrestricted generalization beyond the sampled argumentative texts. They indicate a coordinated reshaping of dispersion, constraint, regularity, and discourse organization within the particular analytic conditions examined here.

## Supplementary Information

Below is the link to the electronic supplementary material.

Supplementary Material 1

Supplementary Material 2

Supplementary Material 3

Supplementary Material 4

Supplementary Material 5

Supplementary Material 6

Supplementary Material 7

Supplementary Material 8

## Data Availability

The public supplementary materials associated with this manuscript include only materials that can be shared under the original consent conditions: derived numeric variables used for the reported analyses, aggregate descriptive and inferential outputs, preprocessing notes, and statistical documentation. Participant-generated essays, text excerpts, source-corpus files, and any other consent-restricted materials have been removed from the public supplementary package and are not publicly available. Because the original consent conditions did not authorize unrestricted public release of learner-generated writing, access to restricted text-level data would require approval from the relevant institutional ethics body, a data-use agreement, and compliance with the original consent conditions. No consent-restricted learner texts are included in the publishable supplementary files.
