# Supplementary material for: Measuring information density in interlanguage through entropy analysis
Source: Sci Rep. 2026 Jun 16;16:18672. doi: 10.1038/s41598-026-56853-3 (PMC13273071; doi:10.1038/s41598-026-56853-3)
Supplement: Supplementary file 1 — Supplementary Material 1 [file 41598_2026_56853_MOESM1_ESM.docx]

**Appendices**

*Supplementary Statistical Tables for the Information-Density Study*

These appendices were revised to match the empirical analytic sample and SPSS outputs. All inferential tests reported here are based on the observed group sizes in the working dataset (B1 = 45, B2 = 55, C1 = 43, L1 = 57; N = 200). To align score direction for omnibus and post-hoc testing, two variables were transformed in SPSS: KLgram_Inv = 7.0 − KLgram and CR_Inv = 7.0 − CR. Thus, higher KLgram_Inv values indicate lower grammatical divergence, and higher CR_Inv values indicate lower original compression-ratio values.

**Appendix Table A1. Analytic sample and operational definitions**

| **Variable** | **Operational definition** | **Direction of higher scores** | **Analytic note** |
| --- | --- | --- | --- |
| Group_A | Observed analytic groups used in inferential testing | — | B1 = 45, B2 = 55, C1 = 43, L1 = 57 |
| Hlex | Lexical entropy | Higher = greater lexical dispersion | Used in original scale |
| PCI | Positional concentration index | Higher = more front-loaded information | Used in original scale |
| IDS_Total | Composite information-density score | Higher = stronger overall information-density profile | Used in original scale |
| KLgram_Inv | Inverse-coded grammatical convergence index | Higher = lower original KLgram | Computed in SPSS as 7.0 − KLgram |
| CR_Inv | Inverse-coded compression regularity index | Higher = lower original CR | Computed in SPSS as 7.0 − CR |

Note. The transformed variables were used to keep the inferential direction consistent across metrics, so that larger values uniformly reflect more advanced performance.

**Appendix Table A2. Descriptive statistics by observed group**

| **Metric** | **Group** | **n** | **Mean** | **SD** | **95% CI** | **Minimum** | **Maximum** |
| --- | --- | --- | --- | --- | --- | --- | --- |
| Hlex | B1 | 45 | 4.131 | 0.087 | [4.105, 4.157] | 3.82 | 4.25 |
| Hlex | B2 | 55 | 4.404 | 0.113 | [4.374, 4.435] | 3.95 | 4.55 |
| Hlex | C1 | 43 | 4.837 | 0.035 | [4.826, 4.847] | 4.75 | 4.95 |
| Hlex | L1 | 57 | 5.202 | 0.148 | [5.163, 5.241] | 4.81 | 5.42 |
| PCI | B1 | 45 | 0.894 | 0.032 | [0.884, 0.903] | 0.81 | 0.94 |
| PCI | B2 | 55 | 1.044 | 0.058 | [1.028, 1.060] | 0.83 | 1.12 |
| PCI | C1 | 43 | 1.191 | 0.017 | [1.186, 1.197] | 1.15 | 1.25 |
| PCI | L1 | 57 | 1.268 | 0.035 | [1.259, 1.277] | 1.18 | 1.35 |
| IDS_Total | B1 | 45 | 4.232 | 0.084 | [4.207, 4.258] | 3.98 | 4.36 |
| IDS_Total | B2 | 55 | 5.022 | 0.277 | [4.947, 5.097] | 4.05 | 5.25 |
| IDS_Total | C1 | 43 | 5.900 | 0.040 | [5.888, 5.913] | 5.82 | 6.05 |
| IDS_Total | L1 | 57 | 6.389 | 0.193 | [6.337, 6.440] | 5.88 | 6.72 |
| KLgram_Inv | B1 | 45 | 5.148 | 0.089 | [5.121, 5.175] | 4.89 | 5.30 |
| KLgram_Inv | B2 | 55 | 5.477 | 0.132 | [5.442, 5.513] | 4.98 | 5.62 |
| KLgram_Inv | C1 | 43 | 6.034 | 0.030 | [6.024, 6.043] | 5.95 | 6.12 |
| KLgram_Inv | L1 | 57 | 6.774 | 0.281 | [6.699, 6.849] | 6.02 | 6.90 |
| CR_Inv | B1 | 45 | 6.338 | 0.016 | [6.333, 6.343] | 6.29 | 6.37 |
| CR_Inv | B2 | 55 | 6.380 | 0.020 | [6.375, 6.385] | 6.30 | 6.40 |
| CR_Inv | C1 | 43 | 6.421 | 0.009 | [6.419, 6.424] | 6.41 | 6.44 |
| CR_Inv | L1 | 57 | 6.474 | 0.022 | [6.468, 6.480] | 6.41 | 6.50 |

Note. Means and confidence intervals are reproduced from the uploaded SPSS descriptive output for the observed analytic sample.

**Appendix Table A3. Shapiro–Wilk normality tests by group**

| **Metric** | **Group** | **W** | **df** | **p** |
| --- | --- | --- | --- | --- |
| Hlex | B1 | 0.907 | 45 | .002 |
| Hlex | B2 | 0.567 | 55 | < .001 |
| Hlex | C1 | 0.904 | 43 | .002 |
| Hlex | L1 | 0.643 | 57 | < .001 |
| PCI | B1 | 0.954 | 45 | .072 |
| PCI | B2 | 0.590 | 55 | < .001 |
| PCI | C1 | 0.915 | 43 | .004 |
| PCI | L1 | 0.818 | 57 | < .001 |
| IDS_Total | B1 | 0.951 | 45 | .057 |
| IDS_Total | B2 | 0.437 | 55 | < .001 |
| IDS_Total | C1 | 0.876 | 43 | < .001 |
| IDS_Total | L1 | 0.631 | 57 | < .001 |
| KLgram_Inv | B1 | 0.973 | 45 | .356 |
| KLgram_Inv | B2 | 0.506 | 55 | < .001 |
| KLgram_Inv | C1 | 0.948 | 43 | .049 |
| KLgram_Inv | L1 | 0.413 | 57 | < .001 |
| CR_Inv | B1 | 0.953 | 45 | .064 |
| CR_Inv | B2 | 0.696 | 55 | < .001 |
| CR_Inv | C1 | 0.840 | 43 | < .001 |
| CR_Inv | L1 | 0.699 | 57 | < .001 |

Note. Several groups deviate from normality, especially for IDS_Total, KLgram_Inv, and CR_Inv. These diagnostics, together with the homogeneity results in Appendix Table A4, support the use of Welch’s ANOVA and Games–Howell pairwise comparisons.

**Appendix Table A4. Homogeneity and robust omnibus tests**

| **Metric** | **Levene F** | **df1** | **df2** | **Welch F** | **Welch df2** | **p** |
| --- | --- | --- | --- | --- | --- | --- |
| Hlex | 6.675 | 3 | 196 | 1181.827 | 98.925 | < .001 |
| PCI | 4.142 | 3 | 196 | 1323.292 | 104.748 | < .001 |
| IDS_Total | 7.817 | 3 | 196 | 5080.653 | 98.326 | < .001 |
| KLgram_Inv | 14.232 | 3 | 196 | 1679.024 | 94.284 | < .001 |
| CR_Inv | 3.306 | 3 | 196 | 503.301 | 104.610 | < .001 |

Note. All five metrics showed significant variance heterogeneity; accordingly, Welch’s one-way ANOVA was retained as the primary omnibus test.

**Appendix Table A5. Games–Howell pairwise comparisons for Hlex**

| **Comparison** | **Mean difference (I − J)** | **SE** | **95% CI lower** | **95% CI upper** | **p** |
| --- | --- | --- | --- | --- | --- |
| B1 vs. B2 | -0.273 | 0.020 | -0.325 | -0.221 | < .001 |
| B1 vs. C1 | -0.705 | 0.014 | -0.742 | -0.669 | < .001 |
| B1 vs. L1 | -1.071 | 0.023 | -1.132 | -1.009 | < .001 |
| B2 vs. C1 | -0.432 | 0.016 | -0.475 | -0.390 | < .001 |
| B2 vs. L1 | -0.798 | 0.025 | -0.862 | -0.733 | < .001 |
| C1 vs. L1 | -0.365 | 0.020 | -0.419 | -0.312 | < .001 |

*Note. All pairwise contrasts remained statistically significant after the Games–Howell correction.*

**Appendix Table A6. Games–Howell pairwise comparisons for PCI**

| **Comparison** | **Mean difference (I − J)** | **SE** | **95% CI lower** | **95% CI upper** | **p** |
| --- | --- | --- | --- | --- | --- |
| B1 vs. B2 | -0.150 | 0.009 | -0.174 | -0.126 | < .001 |
| B1 vs. C1 | -0.297 | 0.005 | -0.312 | -0.283 | < .001 |
| B1 vs. L1 | -0.374 | 0.007 | -0.392 | -0.357 | < .001 |
| B2 vs. C1 | -0.147 | 0.008 | -0.169 | -0.126 | < .001 |
| B2 vs. L1 | -0.224 | 0.009 | -0.248 | -0.200 | < .001 |
| C1 vs. L1 | -0.077 | 0.005 | -0.091 | -0.063 | < .001 |

*Note. All pairwise contrasts remained statistically significant after the Games–Howell correction.*

**Appendix Table A7. Games–Howell pairwise comparisons for IDS_Total**

| **Comparison** | **Mean difference (I − J)** | **SE** | **95% CI lower** | **95% CI upper** | **p** |
| --- | --- | --- | --- | --- | --- |
| B1 vs. B2 | -0.790 | 0.039 | -0.894 | -0.686 | < .001 |
| B1 vs. C1 | -1.668 | 0.014 | -1.705 | -1.631 | < .001 |
| B1 vs. L1 | -2.156 | 0.029 | -2.231 | -2.082 | < .001 |
| B2 vs. C1 | -0.878 | 0.038 | -0.978 | -0.778 | < .001 |
| B2 vs. L1 | -1.367 | 0.045 | -1.485 | -1.248 | < .001 |
| C1 vs. L1 | -0.488 | 0.026 | -0.558 | -0.419 | < .001 |

*Note. All pairwise contrasts remained statistically significant after the Games–Howell correction.*

**Appendix Table A8. Games–Howell pairwise comparisons for KLgram_Inv**

| **Comparison** | **Mean difference (I − J)** | **SE** | **95% CI lower** | **95% CI upper** | **p** |
| --- | --- | --- | --- | --- | --- |
| B1 vs. B2 | -0.329 | 0.022 | -0.388 | -0.271 | < .001 |
| B1 vs. C1 | -0.886 | 0.014 | -0.923 | -0.848 | < .001 |
| B1 vs. L1 | -1.626 | 0.040 | -1.730 | -1.522 | < .001 |
| B2 vs. C1 | -0.556 | 0.018 | -0.605 | -0.508 | < .001 |
| B2 vs. L1 | -1.297 | 0.041 | -1.405 | -1.188 | < .001 |
| C1 vs. L1 | -0.740 | 0.038 | -0.840 | -0.641 | < .001 |

*Note. All pairwise contrasts remained statistically significant after the Games–Howell correction.*

**Appendix Table A9. Games–Howell pairwise comparisons for CR_Inv**

| **Comparison** | **Mean difference (I − J)** | **SE** | **95% CI lower** | **95% CI upper** | **p** |
| --- | --- | --- | --- | --- | --- |
| B1 vs. B2 | -0.042 | 0.004 | -0.052 | -0.033 | < .001 |
| B1 vs. C1 | -0.083 | 0.003 | -0.091 | -0.076 | < .001 |
| B1 vs. L1 | -0.136 | 0.004 | -0.146 | -0.126 | < .001 |
| B2 vs. C1 | -0.041 | 0.003 | -0.049 | -0.033 | < .001 |
| B2 vs. L1 | -0.094 | 0.004 | -0.104 | -0.083 | < .001 |
| C1 vs. L1 | -0.053 | 0.003 | -0.061 | -0.044 | < .001 |

*Note. All pairwise contrasts remained statistically significant after the Games–Howell correction.*
