# Supplementary material for: Measuring information density in interlanguage through entropy analysis
Source: Sci Rep. 2026 Jun 16;16:18672. doi: 10.1038/s41598-026-56853-3 (PMC13273071; doi:10.1038/s41598-026-56853-3)
Supplement: Supplementary file 7 — Supplementary Material 7 [file 41598_2026_56853_MOESM7_ESM.zip › Interlanguage_Supplementary_Statistical_Appendices_Empirical_Sample.docx]

**Appendices**

*Supplementary Statistical Tables for the Information-Density Study*

These appendices report supplementary statistical tables based on the empirical analytic sample and SPSS outputs. All inferential tests reported here are based on the observed group sizes in the working dataset (B1 = 45, B2 = 55, C1 = 43, L1 = 57; N = 200). The four primary variables are reported on their original scales: Hlex, KLgram, CR, and PCI. Hphr is reported as an exploratory descriptive indicator only.

**Appendix Table A1. Analytic sample and operational definitions**

| **Variable** | **Operational definition** | **Direction of higher scores** | **Analytic note** |
| --- | --- | --- | --- |
| Group_Code | Observed empirical analytic groups used in inferential testing | — | B1 = 45, B2 = 55, C1 = 43, L1 = 57 |
| Hlex | Lexical entropy | Higher = greater lexical dispersion | Primary metric |
| KLgram | Grammatical KL divergence from the L1 POS-trigram reference distribution | Lower = closer grammatical alignment with the L1 reference distribution | Primary metric |
| CR | Compression ratio | Lower = greater global structural regularity and compressibility | Primary metric |
| PCI | Positional concentration index | Higher = more front-loaded information | Primary metric |
| Hphr | Entropy over contiguous 3-word lexical sequences | Higher = greater 3-word sequence dispersion | Exploratory descriptive metric only |

*Note. The supplementary tables retain only the variables reported in the manuscript.*

**Appendix Table A2. Descriptive statistics by observed group**

| **Metric** | **Group** | **n** | **Mean** | **SD** | **95% CI** | **Minimum** | **Maximum** |
| --- | --- | --- | --- | --- | --- | --- | --- |
| Hlex | B1 | 45 | 4.131 | 0.087 | [4.105, 4.157] | 3.82 | 4.25 |
| Hlex | B2 | 55 | 4.404 | 0.113 | [4.374, 4.435] | 3.95 | 4.55 |
| Hlex | C1 | 43 | 4.837 | 0.035 | [4.826, 4.847] | 4.75 | 4.95 |
| Hlex | L1 | 57 | 5.202 | 0.148 | [5.163, 5.241] | 4.81 | 5.42 |
| KLgram | B1 | 45 | 1.852 | 0.089 | [1.825, 1.879] | 1.7 | 2.11 |
| KLgram | B2 | 55 | 1.523 | 0.132 | [1.487, 1.558] | 1.38 | 2.02 |
| KLgram | C1 | 43 | 0.966 | 0.03 | [0.957, 0.976] | 0.88 | 1.05 |
| KLgram | L1 | 57 | 0.226 | 0.281 | [0.151, 0.301] | 0.1 | 0.98 |
| CR | B1 | 45 | 0.662 | 0.016 | [0.657, 0.667] | 0.63 | 0.71 |
| CR | B2 | 55 | 0.62 | 0.02 | [0.615, 0.625] | 0.6 | 0.7 |
| CR | C1 | 43 | 0.579 | 0.009 | [0.576, 0.581] | 0.56 | 0.59 |
| CR | L1 | 57 | 0.526 | 0.022 | [0.520, 0.532] | 0.5 | 0.59 |
| PCI | B1 | 45 | 0.894 | 0.032 | [0.884, 0.903] | 0.81 | 0.94 |
| PCI | B2 | 55 | 1.044 | 0.058 | [1.028, 1.060] | 0.83 | 1.12 |
| PCI | C1 | 43 | 1.191 | 0.017 | [1.186, 1.197] | 1.15 | 1.25 |
| PCI | L1 | 57 | 1.268 | 0.035 | [1.259, 1.277] | 1.18 | 1.35 |

*Note. Means and confidence intervals are based on the observed empirical sample.*

**Appendix Table A3. Exploratory descriptive statistics for 3-word lexical-sequence entropy**

| **Group** | **n** | **Mean Hphr** | **SD** | **Sequence type/token ratio** | **Hapax sequences (%)** |
| --- | --- | --- | --- | --- | --- |
| B1 | 45 | 3.21 | 0.42 | 0.88 | 91.2 |
| B2 | 55 | 3.56 | 0.38 | 0.92 | 94.5 |
| C1 | 43 | 3.98 | 0.25 | 0.96 | 97.8 |
| L1 | 57 | 4.32 | 0.31 | 0.98 | 98.2 |

*Note. Hphr = Shannon entropy over contiguous 3-word lexical sequences. Sequence type/token ratio indexes diversity among observed 3-word sequences; hapax sequences are sequences occurring once within the analysis window.*

**Appendix Table A4. Shapiro–Wilk normality tests by group**

| **Metric** | **Group** | **W** | **df** | **p** |
| --- | --- | --- | --- | --- |
| Hlex | B1 | 0.907 | 45 | .002 |
| Hlex | B2 | 0.567 | 55 | < .001 |
| Hlex | C1 | 0.904 | 43 | .002 |
| Hlex | L1 | 0.643 | 57 | < .001 |
| PCI | B1 | 0.954 | 45 | .072 |
| PCI | B2 | 0.59 | 55 | < .001 |
| PCI | C1 | 0.915 | 43 | .004 |
| PCI | L1 | 0.818 | 57 | < .001 |
| KLgram | B1 | 0.973 | 45 | .356 |
| KLgram | B2 | 0.506 | 55 | < .001 |
| KLgram | C1 | 0.948 | 43 | .049 |
| KLgram | L1 | 0.413 | 57 | < .001 |
| CR | B1 | 0.953 | 45 | .064 |
| CR | B2 | 0.696 | 55 | < .001 |
| CR | C1 | 0.84 | 43 | < .001 |
| CR | L1 | 0.699 | 57 | < .001 |

*Note. Several groups deviate from normality; these diagnostics support the use of robust omnibus and post-hoc tests.*

**Appendix Table A5. Homogeneity of variance across groups**

| **Metric** | **Levene F** | **p** |
| --- | --- | --- |
| Hlex | 6.67 | < .001 |
| KLgram | 14.23 | < .001 |
| CR | 3.31 | .021 |
| PCI | 4.14 | .007 |

*Note. Variance homogeneity was violated for the focal metrics, so Welch’s ANOVA and Games–Howell post-hoc tests were retained.*

**Appendix Table A6. Omnibus tests of group differences**

| **Metric** | **Test** | **F** | **eta-squared** | **df** | **p** |
| --- | --- | --- | --- | --- | --- |
| Hlex | Welch ANOVA | 1181.83 | 0.937 | 3, 98.92 | < .001 |
| KLgram | Welch ANOVA | 1679.02 | 0.933 | 3, 94.28 | < .001 |
| CR | Welch ANOVA | 503.3 | 0.889 | 3, 104.61 | < .001 |
| PCI | Welch ANOVA | 1323.29 | 0.929 | 3, 104.75 | < .001 |

*Note. The large effect sizes indicate that proficiency-group membership accounts for a substantial portion of variance in each information-density indicator within this corpus.*

**Appendix Table A7. Games–Howell pairwise comparisons for Hlex**

| **Comparison** | **Mean diff** | **SE** | **t** | **df** | **p** |
| --- | --- | --- | --- | --- | --- |
| B1-B2 | -0.273 | 0.02 | -13.68 | 97.64 | < .001 |
| B1-C1 | -0.705 | 0.014 | -50.55 | 58.23 | < .001 |
| B1-L1 | -1.071 | 0.023 | -45.56 | 92.84 | < .001 |
| B2-C1 | -0.432 | 0.016 | -26.85 | 66.55 | < .001 |
| B2-L1 | -0.798 | 0.025 | -32.12 | 104.4 | < .001 |
| C1-L1 | -0.365 | 0.02 | -17.96 | 63.95 | < .001 |

*Note. Mean diff = mean difference (G1 − G2). Games–Howell pairwise comparisons report approximate t statistics, Welch–Satterthwaite degrees of freedom, and adjusted p values.*

**Appendix Table A8. Games–Howell pairwise comparisons for KLgram**

| **Comparison** | **Mean diff** | **SE** | **t** | **df** | **p** |
| --- | --- | --- | --- | --- | --- |
| B1-B2 | 0.329 | 0.022 | 14.84 | 95.04 | < .001 |
| B1-C1 | 0.886 | 0.014 | 62.77 | 54.28 | < .001 |
| B1-L1 | 1.626 | 0.04 | 41.12 | 69.84 | < .001 |
| B2-C1 | 0.556 | 0.018 | 30.35 | 61.15 | < .001 |
| B2-L1 | 1.297 | 0.041 | 31.45 | 80.05 | < .001 |
| C1-L1 | 0.74 | 0.038 | 19.74 | 57.71 | < .001 |

*Note. Mean diff = mean difference (G1 − G2). Games–Howell pairwise comparisons report approximate t statistics, Welch–Satterthwaite degrees of freedom, and adjusted p values.*

**Appendix Table A9. Games–Howell pairwise comparisons for CR**

| **Comparison** | **Mean diff** | **SE** | **t** | **df** | **p** |
| --- | --- | --- | --- | --- | --- |
| B1-B2 | 0.042 | 0.004 | 11.61 | 98.0 | < .001 |
| B1-C1 | 0.083 | 0.003 | 30.18 | 66.83 | < .001 |
| B1-L1 | 0.136 | 0.004 | 35.38 | 99.45 | < .001 |
| B2-C1 | 0.041 | 0.003 | 13.75 | 76.62 | < .001 |
| B2-L1 | 0.094 | 0.004 | 23.36 | 109.34 | < .001 |
| C1-L1 | 0.053 | 0.003 | 16.21 | 75.76 | < .001 |

*Note. Mean diff = mean difference (G1 − G2). Games–Howell pairwise comparisons report approximate t statistics, Welch–Satterthwaite degrees of freedom, and adjusted p values.*

**Appendix Table A10. Games–Howell pairwise comparisons for PCI**

| **Comparison** | **Mean diff** | **SE** | **t** | **df** | **p** |
| --- | --- | --- | --- | --- | --- |
| B1-B2 | -0.15 | 0.009 | -16.42 | 86.23 | < .001 |
| B1-C1 | -0.297 | 0.005 | -55.08 | 68.99 | < .001 |
| B1-L1 | -0.374 | 0.007 | -56.6 | 98.24 | < .001 |
| B2-C1 | -0.147 | 0.008 | -17.82 | 65.92 | < .001 |
| B2-L1 | -0.224 | 0.009 | -24.62 | 88.14 | < .001 |
| C1-L1 | -0.077 | 0.005 | -14.39 | 86.14 | < .001 |

*Note. Mean diff = mean difference (G1 − G2). Games–Howell pairwise comparisons report approximate t statistics, Welch–Satterthwaite degrees of freedom, and adjusted p values.*
