# Supplementary material for: Measuring information density in interlanguage through entropy analysis
Source: Sci Rep. 2026 Jun 16;16:18672. doi: 10.1038/s41598-026-56853-3 (PMC13273071; doi:10.1038/s41598-026-56853-3)
Supplement: Supplementary file 8 — Supplementary Material 8 [file 41598_2026_56853_MOESM8_ESM.docx]

**Appendix A**

**Corpus Dataset of Participant Argumentative Essays**

**Corpus summary.** The appendix presents the de-identified L2 argumentative essays organized by proficiency level. The corpus comprises B1 essays (n = 50), B2 essays (n = 50), and C1 essays (n = 50), for a total of 150 essays. Original grouping labels have been removed, and essays are ordered sequentially within each proficiency group.

**Table A1**

*Corpus composition by proficiency level*

| **Proficiency level** | **Number of essays** | **Description** |
| --- | --- | --- |
| B1 | 50 | Intermediate learner essays |
| B2 | 50 | Upper-intermediate learner essays |
| C1 | 50 | Advanced learner essays |

# Level B1 (Intermediate)

## Essay B1-1. Is online learning better than classroom learning?

In my opinion, I think online learning is very good for many people in Egypt. First, it is very cheap because you don't need to travel to the university. You can stay at home and watch the teacher on the computer. This is good for students who live in small villages and don't have much money. Second, you can watch the video many times. If you don't understand the lesson, you can play it again. This helps me a lot in my studies. But, some people say that online learning is bad because we don't see our friends. I think this is true, but we can talk to them on WhatsApp or Facebook. Also, sometimes the internet is very slow in some places, and this is a big problem. In conclusion, online learning has some problems but it is better than classroom learning for many students because it is easy and saves time.

## Essay B1-2. The importance of tourism for Egypt.

Tourism is very important for the Egyptian economy. Every year, many tourists come to Egypt to see the Pyramids and the Sphinx. This is very good because tourists spend a lot of money in our country. They stay in hotels, eat in restaurants, and buy many things from the markets. This gives jobs to many young people in Egypt. For example, my brother works in a hotel in Sharm El-Sheikh. He likes his job very much. Also, when tourists come, they learn about our culture and history. We should be very kind to them so they come back again. Some people are not nice to tourists, and this is a bad thing for our country. We must protect the tourists and keep our cities clean. In conclusion, I believe that tourism is the most important thing for our future and we should help it to grow.

## Essay B1-3. Living in a big city vs. a small village.

Some people like to live in big cities like Cairo, but other people like to live in small villages. In my opinion, living in a big city is better. First, there are many shops, hospitals, and schools. If you are sick, you can find a good doctor very fast. Also, there are many jobs in the city. In the village, most people work in farms and they don't have much money. Second, the city is very exciting. You can go to the cinema, the park, or the mall with your friends. But the city is also very noisy and there is a lot of traffic. Sometimes it is difficult to breathe because of the pollution. The village is quiet and the air is clean, but it is very boring for young people. Finally, I think the city is the best place to live because it has everything you need for a good life.

## Essay B1-4. Should students wear uniforms at school?

I think all students should wear uniforms at school. There are many reasons for this. First, when everyone wears the same clothes, the students look very neat and organized. The teachers like this. Second, it makes all students equal. In my school, some students are rich and some are poor. If we don't have a uniform, the rich students will wear expensive clothes and the poor students will feel sad. This is not fair. Also, uniforms save a lot of time in the morning. You don't have to think about what you will wear today. You just put on the uniform and go to school. Some students say that uniforms are ugly and they want to wear their own clothes. But I think school is for learning, not for fashion. In conclusion, school uniforms are a good idea because they make students equal and save time.

## Essay B1-5. The effects of fast food on health.

Nowadays, many people eat fast food every day because they are very busy. They go to restaurants like McDonald's or KFC. In my opinion, fast food is very bad for our health. First, it has a lot of fat and sugar. This makes people very fat and they get diseases like heart problems or diabetes. This is a big problem in Egypt now. Second, fast food is not fresh. It is made a long time ago and then heated up. This is not good for the stomach. Also, it is very expensive. If you cook at home, it is cheaper and more healthy. For example, my mother cooks delicious vegetables and rice every day. I feel very strong when I eat her food. In conclusion, we should stop eating fast food and start eating healthy food at home to protect our bodies and save our money.

## Essay B1-6. Why learning English is important.

In my opinion, learning English is very important for all people in the world today. First, English is a global language. If you speak English, you can travel to any country and talk to people easily. For example, if I go to London or New York, I can ask for directions and buy food. Second, English is the language of science and technology. Most of the information on the internet is in English. If a student wants to learn about computers or medicine, he must know English very well. Also, English helps you to get a good job. Many big companies in Egypt want workers who can speak and write in English. If you have good English, your salary will be higher. Some people say English is difficult to learn, but I think if you practice every day, it becomes easy. You can watch movies and listen to songs. In conclusion, learning English is the key to a better future and a good career.

## Essay B1-7. Is the internet good or bad for children?

Many children use the internet every day for playing games and watching videos. I think the internet is good for children, but it can also be bad. First, the internet is very helpful for school. Children can find information for their homework very fast. They can learn about history, science, and geography from websites like YouTube and Wikipedia. This makes learning fun for them. Second, children can talk to their friends and family on the internet. This is good for their social life. However, there are some problems. Some children spend too many hours on the computer and they don't do any sports. This makes them unhealthy and fat. Also, there is some bad information on the internet that is not for children. Parents should watch their children when they use the internet. In conclusion, the internet is a very useful tool, but children must use it carefully and not spend all their time on it.

## Essay B1-8. The benefits of shopping in big malls.

Nowadays, many people in Egypt like to go to big shopping malls like Mall of Arabia or City Stars. In my opinion, shopping in malls is better than shopping in small markets. First, malls are very comfortable. They have air conditioning, so you don't feel hot in the summer. You can walk for a long time and not feel tired. Second, you can find everything in one place. Malls have clothes, shoes, electronics, and food. You don't need to go to many different streets to buy what you need. This saves a lot of time and effort. Also, malls have cinemas and play areas for children. You can go with your family and have a fun day. But, some people say malls are very expensive and they prefer small shops. I think this is true sometimes, but malls often have big sales. In conclusion, shopping malls are the best place for shopping and entertainment for modern families.

## Essay B1-9. My favorite hobby: Reading books.

Everyone should have a hobby to enjoy their free time. My favorite hobby is reading books. I like reading very much because it makes me feel happy and relaxed. First, reading gives me a lot of information. When I read a book about history or science, I learn many new things about the world. This helps me in my school and my life. Second, reading improves my language. When I read English books, I learn new words and my grammar becomes better. Also, reading takes me to different places. I can sit in my room in Cairo and read about life in Japan or the North Pole. This is like magic. Some of my friends like playing football or video games, but I prefer sitting with a good book. It is a quiet and cheap hobby. In conclusion, I believe that reading is the best hobby because it develops the mind and makes us more intelligent.

## Essay B1-10. The importance of sports for health.

Doing sports is very important for a healthy life. Many people today sit at desks for a long time and they don't move. In my opinion, everyone should do sports at least three times a week. First, sports make your body strong. When you run, swim, or play football, your heart and muscles become healthy. This prevents many diseases like heart attacks. Second, sports are good for the mind. When I play sports with my friends, I feel happy and I forget my problems. It reduces stress from school and work. Also, sports teach us many things like teamwork and discipline. You learn how to follow rules and work with other people to win. For example, football is very popular in Egypt and it brings people together. In conclusion, we should all practice sports to keep our bodies and minds healthy and to enjoy our time with friends.

## Essay B1-11. Is it better to be an only child or have siblings?

In my opinion, I think it is much better to have brothers and sisters than to be an only child. First, if you have siblings, you are never lonely. You always have someone to play with and talk to at home. For example, I have two brothers and we play football together every day in the street. This makes me very happy. Second, siblings help each other. If I have a problem with my homework, my big sister can explain it to me. We also share our toys and our clothes. Some people say that being an only child is good because you get all the toys and all the money from your parents. But I think this is boring because you don't have anyone to share your life with. Also, when you grow up, your siblings will help you in difficult times. In conclusion, having a big family is a great gift and it teaches us how to love and help other people.

## Essay B1-12. The role of computers in schools.

Nowadays, every school in Egypt should have many computers for the students. I think computers are very important for education. First, students can learn many things very fast. They can use the internet to look for information about history or science. This is better than just reading the old books in the library. Second, computers help students to be more creative. They can learn how to draw, make videos, or write stories on the computer. This is very useful for their future jobs because every company now uses computers. Also, teachers can use the computer to show beautiful pictures and videos to the class. This makes the lesson very interesting. Some people say that computers are bad because students play games and don't study. I think the teacher must watch the students and help them to use the computer in a good way. In conclusion, computers are the most important tool for modern schools and they help us to learn better.

## Essay B1-13. Why people like to travel to new places.

Many people save money all year because they want to travel to a new place or a different country. In my opinion, travel is very good for everyone. First, you can see many beautiful things. For example, if you go to Aswan, you can see the Nile and the old temples. If you go to France, you can see the Eiffel Tower. This makes life very exciting. Second, you can learn about different cultures and eat new food. I like to try different food when I go to a new city. It is very delicious. Also, travel helps you to relax and forget about the stress of work and school. You can sit on the beach or walk in the mountains. Some people say travel is too expensive and a waste of money. But I think the memories of travel are more important than money. In conclusion, traveling is a great experience because it opens our minds and makes us happy.

## Essay B1-14. Is watching television bad for children?

Many children watch television for many hours every day. I think television can be bad for children if they watch it too much. First, it is bad for their eyes. If a child sits very close to the TV for a long time, his eyes will be very tired and he may need glasses. Second, television makes children lazy. Instead of playing outside or doing sports, they just sit on the sofa and eat snacks. This makes them fat and unhealthy. Also, some movies on TV are very violent and children might learn bad behavior from them. However, there are some good programs like cartoons that teach English or documentaries about animals. These can be very helpful for learning. But parents must choose the programs and limit the time. In conclusion, watching TV is okay for a short time, but children should spend more time playing and studying to be healthy and smart.

## Essay B1-15. The importance of recycling for the environment.

We must protect our environment because it is our home. I think recycling is a very good way to keep our country clean. First, recycling saves our natural resources. When we recycle paper, we don't need to cut down many trees. When we recycle plastic bottles, we can make new things from them. This is very good for the earth. Second, recycling reduces the rubbish in the streets. In some cities in Egypt, there is a lot of trash and this smells bad and brings diseases. If we put the plastic, glass, and paper in different bins, the streets will be very beautiful and clean. Also, recycling can give jobs to many people who work in the factories. Some people think recycling is difficult because they are lazy. But I think it is very easy to have two bins at home. In conclusion, we should all start recycling today to save our planet and live in a clean world.

## Essay B1-16. Should we spend money on space exploration?

In my opinion, I think spending money on space exploration is not a good idea for now. There are many reasons for this. First, we have many problems on the Earth that need money. For example, in many countries, there are people who are very poor and don't have food or clean water. Also, we need more hospitals and better schools. If we spend billions of dollars on rockets to Mars, we are wasting money that can help people here. Second, space is very dangerous and difficult. I think we should fix our own planet first before we go to other planets. Some people say that space exploration is good for science and new technology. I agree that science is important, but the lives of people are more important. We should protect the environment and stop the climate change on Earth. In conclusion, I believe the government should spend money on the poor people and the education instead of sending robots to space.

## Essay B1-17. The importance of family in our life.

Family is the most important thing in the world for every person. In my opinion, without family, life is very difficult and sad. First, your family always loves you and helps you. When I have a problem in my school or with my friends, I talk to my mother and father. they give me good advice and make me feel better. My brothers also play with me and we have a lot of fun together. Second, family teaches us how to be a good person. We learn how to respect old people and how to be kind to others from our parents. In Egypt, family is very big and we always visit our grandparents and cousins on the weekends. This makes the social life very strong. Some people like to live alone, but I think this is very lonely. You need someone to share your happy and sad moments. In conclusion, family is a great blessing from God and we should always take care of our family members.

## Essay B1-18. Should students use cell phones in the classroom?

Nowadays, almost every student has a smartphone. Some teachers say that students should not use phones in the class, but I disagree. I think cell phones can be very useful for learning. First, students can use the internet to find information quickly. If the teacher says a difficult word, I can look it up in the dictionary on my phone. Also, we can take photos of the whiteboard so we don't have to write everything very fast. This helps us to understand the lesson better. Second, phones are important for safety. If there is an emergency, the student can call his parents immediately. However, I know that some students play games or talk to friends on WhatsApp during the lesson. This is a bad thing. The teacher should make some rules for using the phone. For example, we only use it when the teacher says "yes." In conclusion, cell phones are a great tool for education if we use them in a correct and responsible way.

## Essay B1-19. The importance of water for life.

Water is the most important thing for all living things. Without water, humans, animals, and plants will all die. In my opinion, we must save water every day. First, water is necessary for our health. We need to drink a lot of water to keep our bodies strong and to clean our skin. Also, we use water for cleaning our houses and washing our clothes. In Egypt, the Nile River is the main source of water, and it is very important for our history and our economy. Second, farmers need water to grow food. If there is no water, we will not have vegetables or fruits to eat. This is a big problem for the world. Some people waste water when they wash their cars or brush their teeth. This is very bad behavior. We should use only a small amount of water and turn off the tap. In conclusion, water is life, and we should protect the Nile and all our water sources for the next generation.

## Essay B1-20. Should exams be replaced by projects?

Many students feel very stressed when they have exams. In my opinion, I think that projects are better than exams for testing the students. First, projects show what a student can really do. When I make a project about history, I read many books, find pictures, and make a presentation. I learn more things this way than just memorizing facts for an exam. Exams only test the memory, but projects test the creativity. Second, exams make students very nervous and sometimes they forget everything because they are afraid. This is not fair because some students are very clever but they don't like exams. Projects give students more time to think and work carefully at home. However, some people say that students might ask their parents to do the project for them. This is a problem, but the teacher can talk to the student to check his work. In conclusion, I believe that projects are a more modern and fair way to help students learn and grow.

## Essay B1-21. Why some people prefer living in the city.

Many people in Egypt like to move from the village to the big city like Cairo. In my opinion, there are many reasons for this. First, the city has many jobs. If you are a young man and you want to earn a lot of money, you can find a job in a factory or a big office in the city. In the village, there is only farming. Second, the city has many schools and big universities. This is very good for the students who want to have a good education and a better future. Also, the city is very fun at night. You can go to the mall, the restaurant, or the park with your family. However, the city is very crowded and there is a lot of traffic. Sometimes it takes two hours to go to work. But I still think the city is better because it has everything you need for a modern life. In conclusion, living in the city is exciting and has many opportunities for everyone.

## Essay B1-22. The importance of learning a new language.

I think that learning a new language like English or French is very useful for every person today. First, it helps you to talk to people from different countries. If you know English, you can make friends on the internet and learn about their lives. This is very interesting. Second, a new language is very important for your career. If you want to work in a big company in Egypt, you must speak English well. They will give you more money if you have good language skills. Also, you can travel to many places and not feel afraid because you can ask for help in English. Some people say that learning a language is very hard and takes a long time. I think this is true, but we should try our best. You can use apps on your phone to practice every day. In conclusion, learning a new language opens many doors and helps us to understand the world better.

## Essay B1-23. Is it good to have a part-time job as a student?

Some students work in cafes or shops after they finish their school. In my opinion, I think having a part-time job is a very good thing for students. First, it gives them money. Students can buy their own books, clothes, and phones without asking their parents for money. This makes them feel independent and happy. Second, a job teaches students about responsibility. They learn how to be on time and how to talk to customers in a polite way. This is a very good experience for their life after university. However, some parents think that a job will make the student tired and he will not study well. I think this is a problem, so the student should only work for a few hours. He must put his education first. In conclusion, a part-time job is good because it teaches students how to work and helps them to be more mature.

## Essay B1-24. The role of teachers in our society.

Teachers are the most important people in our society because they help children to learn. In my opinion, we should respect teachers very much. First, teachers give us knowledge. They teach us how to read, write, and do math. Without teachers, we will not have doctors, engineers, or scientists. They are like a second father or mother to the students. Second, teachers teach us good manners. They tell us how to be honest, kind, and how to help others. In Egypt, teachers work very hard for many hours in the school. Sometimes the class is very big and noisy, but the teacher is patient and helps everyone. Also, teachers encourage us to follow our dreams. Some people say that being a teacher is a difficult job with a small salary. I think the government should give them more money because they build the future. In conclusion, teachers are very special and they deserve our love and respect.

## Essay B1-25. Should the government build more parks in the city?

I believe that the government should build more green parks in every city in Egypt. There are many reasons for this. First, parks are very good for the health. People can go there to walk, run, or do some sports in the fresh air. This is better than staying at home and watching TV. Second, parks are a great place for families to spend time together. On the weekends, parents can take their children to play in the grass and have a picnic. It is a very cheap and happy way to enjoy the holiday. Also, trees in the parks help to clean the air from the pollution of the cars. This makes the city more beautiful and healthy. Some people say that we need more buildings and houses instead of parks. I think houses are important, but we also need nature to feel relaxed. In conclusion, more parks will make our cities better and our people more healthy and happy.

## Essay B1-26. Is it better to travel alone or with a group?

In my opinion, I think it is better to travel with a group of friends or family than to travel alone. First, traveling in a group is much safer. If you are in a new country and you get lost or you have a problem, your friends can help you. You don't feel afraid because you are not alone. Second, it is more fun to share the experience. When you see a beautiful place like the Eiffel Tower, you can take photos together and talk about it. This makes the trip very happy. Also, traveling in a group is often cheaper. You can share the price of the hotel room and the taxi. Some people say traveling alone is good because you can do whatever you want. I think this is true, but it can be very lonely at night when you eat dinner alone. In conclusion, traveling with a group is the best choice because it is safe, cheap, and you can share the joy with the people you love.

## Essay B1-27. Is it better to be rich or famous?

Many people dream about being rich or being a famous star on television. In my opinion, it is much better to be rich than to be famous. First, if you are rich, you can buy everything you need. You can have a big house, a fast car, and travel to any country. You can also help your family and give money to poor people in Egypt. This is a very good thing. Second, being famous is very difficult for the personal life. If you are a famous actor, people follow you everywhere and take photos of you. You don't have any privacy. You cannot go to the restaurant or the park with your children easily. This makes the famous person very stressed and sad. Also, some people might say bad things about you on the internet. In conclusion, money is better because it gives you a comfortable life and freedom, but fame takes away your freedom and makes you tired.

## Essay B1-28. The importance of a good diet for students.

I think that a healthy diet is very important for all students to study well. First, healthy food like fruits and vegetables gives the brain more energy. When I eat a good breakfast with eggs and milk, I can understand the lesson very fast in the morning. I don't feel tired or sleepy in the class. Second, a good diet protects us from diseases. If we eat too much sugar and fast food, we will become fat and sick. This means we will miss many days of school. Also, drinking a lot of water is very important for the concentration. Some students drink a lot of coffee or soda, but I think this is bad for the nerves. My mother always tells me to eat fish and nuts because they are good for the memory. In conclusion, students should eat healthy food to keep their bodies strong and to get high marks in their exams.

## Essay B1-29. Should we have a long summer holiday?

In Egypt, the summer holiday is very long, about three months. Some people think this is good, but others think it is too long. In my opinion, a long holiday is a great thing for students and teachers. First, students need a long time to rest after a hard year of studying. They have a lot of stress from exams and homework, so they need to relax and play. They can go to the beach in Alexandria or Marsa Matrouh. Second, a long holiday is a good time to learn new hobbies. Students can learn how to swim, draw, or play a musical instrument. This is good for their personality. However, some people say that students forget their lessons during the long holiday. I think they can read some books for one hour every day so they don't forget everything. In conclusion, a long summer holiday is necessary for the health and happiness of the students.

## Essay B1-30. The importance of learning history.

Many students think that history is a boring subject because they have to memorize many dates. In my opinion, learning history is very important for every person. First, history teaches us about our country and our identity. When we learn about the ancient Egyptians and the great Kings, we feel very proud of our history. We understand how people lived in the past. Second, history helps us to not make the same mistakes again. If we know about the old wars and the problems of the past, we can have a better future. Also, history is full of great stories about brave people. This can encourage young people to be strong and work hard for their country. Some people say that we should only study science and math. I think science is important, but history is the soul of the nation. In conclusion, history is an interesting subject that helps us to understand the world and ourselves.

## Essay B1-31. Is it better to be a leader or a follower?

In my opinion, I think it is better to be a leader than to be a follower. First, a leader can make his own decisions. He can choose the best way to do a project in school or a job in a factory. This makes the leader feel very confident and strong. Second, a leader helps other people. A good leader listens to his friends and gives them advice. For example, if we are playing football, the captain tells us how to win the game. This is a very important role. However, some people say that being a leader is very stressful because everyone looks at you when there is a problem. I think this is true, but it is also exciting. Followers are also important because they do the work, but they don't have much power. I want to be a leader in the future to help my country and my family. In conclusion, being a leader is the best choice for people who want to be successful and help others.

## Essay B1-32. The impact of tourism on historical sites.

Egypt has many historical sites like the Pyramids and the temples in Luxor. Tourism is very good for these places, but it can also be bad. First, tourism brings a lot of money. The government can use this money to fix the old stones and clean the temples. This keeps our history safe for a long time. Second, tourism gives many jobs to the people who live near the historical sites. They can work as tour guides or sell souvenirs. But, there are some problems. Many tourists walk on the old monuments and sometimes they touch the walls. This can damage the ancient paintings. Also, some people throw rubbish in the area, which makes the place look bad. We must tell the tourists to follow the rules and protect our history. In conclusion, tourism is very important for our economy, but we must take care of our historical sites so they stay beautiful for the next generation.

## Essay B1-33. Should we ban cars in city centers?

In my opinion, I think banning cars in the city center is a very good idea. First, it will reduce the pollution. In big cities like Cairo, there are too many cars and the air is very dirty. If we stop the cars, the air will be fresh and people can breathe better. This is good for the health. Second, it will be safer for people to walk. Sometimes it is very difficult to cross the street because the cars are very fast. If there are no cars, children and old people can walk easily and enjoy the shops. Also, it will be very quiet and peaceful. However, some shop owners say that they will lose money because people cannot come by car. I think they can use the metro or the bus to reach the center. In conclusion, banning cars will make our cities more beautiful, healthy, and safe for every person.

## Essay B1-34. The importance of learning about other cultures.

I think that learning about other cultures is very important for all people today. First, it helps us to understand how other people live. For example, when I learn about life in Japan or America, I see that they have different food and different traditions. This makes me more open-minded. Second, it helps to stop problems between countries. If we know about each other, we will not have wars or hate. We will see that all people are similar in some ways. Also, learning about culture is very fun. You can try new food, listen to new music, and learn new stories. In Egypt, we have many tourists from different cultures, and we should be kind to them and learn from them. In conclusion, the world is like a big family, and learning about other cultures is the only way to live together in peace and happiness.

## Essay B1-35. Should homework be abolished?

Many students hate doing homework after a long day at school. In my opinion, I think homework should be abolished or at least be very small. First, students are very tired. They spend seven hours in the school and then they have to study for three more hours at home. This is too much and it makes them feel stressed and sad. They don't have time to play or see their friends. Second, students should learn everything inside the classroom with the teacher. If a student doesn't understand the lesson in school, he will not be able to do the homework alone at home. This makes the student feel stupid. However, some teachers say that homework is good for practice. I think one or two questions is enough, not many pages. In conclusion, abolishing homework will make students more happy and give them time to enjoy their childhood.

## Essay B1-36. Why team sports are good for children.

In my opinion, I think that team sports like football or basketball are very good for all children. First, team sports help children to make many friends. When they play together in a team, they talk and help each other to win the game. This makes them very happy and they don't feel lonely. Second, children learn about teamwork. In life, we need to work with other people in the office or in the family. Team sports teach us how to follow the rules and how to listen to the captain. This is a very important lesson for the future. Also, sports keep the body healthy and strong. In Egypt, football is the most popular sport and many children dream to be like Mohamed Salah. They work hard and stay away from bad things like smoking. In conclusion, team sports are the best way for children to be healthy and to learn how to live with others in a good way.

## Essay B1-37. Is it better to work during the day or at night?

Some people like to work at night because it is quiet, but in my opinion, it is much better to work during the day. First, the day is the natural time for work. When the sun is out, our bodies have a lot of energy and we can think very fast. If you work at night, you feel very sleepy and tired. This is bad for the health and the heart. Second, the day is better for the social life. If you work during the day, you can see your family and your friends in the evening. You can have dinner together and watch TV. If you work at night, you sleep all the day and you don't see anyone. This makes the person feel very lonely and sad. Also, most offices and shops are open during the day, so it is easier to finish your business. In conclusion, working during the day is the best choice for a healthy and happy life.

## Essay B1-38. The importance of English for travel.

I believe that English is the most important language if you want to travel to different countries. First, English is a global language. In every airport, hotel, and restaurant in the world, people speak English. If you have a problem or you lose your way, you can ask anyone for help in English and they will understand you. This makes you feel safe when you are far from home. Second, English helps you to learn about the culture of the place. You can talk to the local people and understand their traditions and their history. For example, if I go to Japan, I don't speak Japanese, but I can talk to Japanese people in English. This is like a bridge between people. Also, you can read the signs and the menus easily. In conclusion, learning English is the key to a successful trip and it helps us to see the world without any fear.

## Essay B1-39. Living in a house vs. an apartment.

Many people in Egypt live in apartments in big buildings, but some people prefer to live in a big house with a garden. In my opinion, living in a house is much better. First, a house has more space and privacy. You don't have neighbors above you or below you making noise. You can play music or talk loudly and no one will be angry. This makes you feel free and relaxed. Second, a house usually has a garden. Children can play in the grass and you can grow some flowers or vegetables. This is very good for the health and the fresh air. However, an apartment is often cheaper and closer to the city center and the shops. This saves a lot of money and time. But I think the comfort of a house is more important than money. In conclusion, living in a house is the best way to have a happy and quiet family life.

## Essay B1-40. The influence of movies on young people.

Nowadays, young people watch many movies on Netflix or in the cinema. In my opinion, movies have a big influence on their behavior and their ideas. First, movies can teach young people good things. For example, some movies show stories about brave people who help their country or their friends. This encourages the youth to be kind and strong. Also, movies are a great way to learn a new language and see how people live in other countries. Second, some movies can be bad. They show a lot of violence and smoking, and some teenagers might try to imitate these bad things. This is a big problem for the society. Parents should watch the movies with their children and talk about what is right and what is wrong. In conclusion, movies are a powerful tool that can be used for good or for bad, and we should choose the movies we watch very carefully.

## Essay B1-41. The importance of learning to cook.

In my opinion, I think that every young person should learn how to cook their own food. First, cooking at home is much healthier than eating fast food. When you cook, you can choose fresh vegetables and good meat, and you don't put too much salt or oil. This is very good for the heart and the body. Second, learning to cook saves a lot of money. Buying food from restaurants every day is very expensive for a student or a young worker. If you go to the market and buy rice, pasta, and vegetables, you can eat for many days with a small amount of money. Also, cooking is a very useful skill for the life. For example, if I live alone in a new city, I can take care of myself and be independent. Some people say that cooking takes a long time and it is boring. I think if you listen to music and try new recipes, it becomes a very fun hobby. In conclusion, cooking is an essential skill that helps us to be healthy, save money, and feel proud of ourselves.

## Essay B1-42. Is it better to spend money or save it?

Many people like to spend all their money on clothes, phones, and going out. But in my opinion, it is much better to save some money for the future. First, you never know what will happen tomorrow. If you have an emergency, like if you get sick or your car breaks down, you need money to fix the problem quickly. If you spend everything, you will have to ask people for help and this is very stressful. Second, saving money helps you to reach your big goals. For example, if I want to buy a house or get married in the future, I must start saving now. This makes me feel safe and responsible. However, I think we should not be very cheap. It is okay to spend some money on fun things sometimes to be happy. But the most important thing is to have a balance. In conclusion, saving money is a very good habit that gives us a comfortable life and protects us from the difficult times in the future.

## Essay B1-43. Why we should plant more trees in our streets.

I believe that the government and the people should work together to plant more trees in all the cities in Egypt. First, trees make the air very clean. They take the bad air from the cars and give us fresh oxygen. In a crowded city like Cairo, we need many trees to breathe better and to stay healthy. Second, trees make the city look beautiful and green. When I walk in a street with many trees, I feel very relaxed and happy. It is much better than looking at only concrete and gray buildings. Also, trees give us shade in the summer. The sun in Egypt is very hot, and the trees make the temperature lower for the people who are walking. Some people think that trees take a lot of water. I think we can use recycled water to keep them green. In conclusion, planting trees is a simple and cheap way to make our life better and to protect our environment for the next generation.

## Essay B1-44. Should children have their own tablets?

Nowadays, many children have their own tablets to play games and watch videos. I think this can be good, but it can also be very bad for the child. First, tablets are good for learning. There are many apps that teach children how to draw, speak English, or solve math problems. This makes the child very smart and helps him in the school. Second, tablets keep the children quiet and busy when the parents are doing work. However, there are many problems. If a child uses the tablet for a long time, he will not play with his friends or do any sports. This makes him very lazy and unhealthy. Also, some things on the internet are not good for children, so the parents must watch them very carefully. In my opinion, children can use the tablet for only one hour a day. In conclusion, tablets are a good tool for education, but we should not let them take the place of real play and social life.

## Essay B1-45. The importance of friendship in our life.

Friends are very important for every person because they make life more beautiful and fun. In my opinion, we cannot live happily without good friends. First, friends help us in the difficult times. When I am sad or I have a problem in my studies, I talk to my best friend. He listens to me and gives me good advice. This makes me feel that I am not alone. Second, we share our happy moments with our friends. When I have a birthday or I get a high mark in the exam, I celebrate with my friends. We laugh and have a great time together. These are the best memories in life. To have a good friend, you must be a good friend too. You should be honest, kind, and help them when they need you. In conclusion, friendship is a great gift, and we should take care of our friends and always stay close to them because they are like our second family.

## Essay B1-46. Is online shopping better than traditional shopping?

Nowadays, many people use their phones to buy clothes and food from websites like Amazon or Noon. In my opinion, online shopping is very good but it has some problems. First, it is very convenient and saves time. You don't have to go to the shop and wait in the traffic. You can buy anything you want while you are sitting on your sofa. This is very good for busy people. Second, you can compare the prices very fast and find the cheapest things. However, sometimes the clothes you buy don't look like the picture on the website. Maybe the size is too small or the color is different. This is very annoying because you have to send it back. Also, you have to wait for many days for the delivery. In conclusion, I like online shopping for electronics and books, but for clothes, I think it is better to go to the real shop to try them on.

## Essay B1-47. The importance of music in our lives.

I think that music is a very important part of life for everyone. First, music helps us to relax. When I am stressed from my school or my exams, I listen to some quiet music and I feel much better. It is like a medicine for the soul. Second, music brings people together. At weddings or parties in Egypt, people dance and sing together. This makes a very happy atmosphere. Also, music can help you to learn a new language. I listen to English songs and I learn many new words and how to pronounce them. This is a very fun way to study. Some people say that music is a waste of time and students should only study. I disagree with this because the brain needs some rest and joy to work better. In conclusion, life without music would be very boring and sad, and we should all enjoy it every day.

## Essay B1-48. Should we keep animals in our homes?

Many people like to have pets like dogs, cats, or birds in their houses. In my opinion, having an animal at home is a very good thing, especially for children. First, pets teach children about responsibility. The child must give food to the animal and clean it every day. This helps the child to be more mature and kind. Second, animals are very good friends. If you live alone, a cat or a dog can make you feel happy and not lonely. They always love you and wait for you at the door. However, having a pet is also a lot of work and it costs a lot of money for the food and the doctor. You must keep your house very clean because animals can bring some dirt. In conclusion, I believe that pets are a great gift for the family if you have the time and the money to take care of them.

## Essay B1-49. How will life be in the future?

I think that life in the future will be very different because of new technology and robots. First, I believe that we will have flying cars. This will solve the problem of traffic in big cities like Cairo. People will move very fast from one place to another. Second, robots will do all the difficult and boring work. They will clean the houses, build the buildings, and maybe they will even cook our food. This will give humans more time to play and travel. Also, I think we will use only clean energy from the sun. However, I am worried that people will be very lazy and they will not talk to each other in real life. They will spend all their time in virtual reality. In conclusion, the future will be very exciting and easy, but we must stay healthy and keep our social life strong.

## Essay B1-50. The importance of being polite.

In my opinion, being polite is the most important quality for any person. If you are polite, everyone will respect you and like to talk to you. First, being polite helps to avoid problems with people. If you say "please" and "thank you" and speak in a quiet voice, people will be kind to you. In the school or in the street, we should always help the old people and the children. This makes our society very peaceful and happy. Second, polite people are more successful in their jobs. A boss likes a worker who is respectful and has good manners. If you are rude, you will lose your friends and your job very fast. In Egypt, we learn from our parents to respect the teachers and the family. In conclusion, manners are the most beautiful thing a person can have, and they make the world a better place for everyone.

# Level B2 (Upper-Intermediate)

## Essay B2-1. Should the government invest more in public transport?

The question of whether the government should prioritize investment in public transport is a significant issue in rapidly developing countries like Egypt. In my opinion, improving the public transport system is essential for economic growth and environmental sustainability. Firstly, better public transport, such as expanding the metro lines in Cairo, would significantly reduce traffic congestion. Currently, many employees spend hours commuting to work, which reduces their productivity. If the metro reached more areas, people would leave their cars at home. Secondly, it is a more environmentally friendly option. Reducing the number of private vehicles on the road would decrease air pollution, which is a major health concern in our capital. However, some argue that these projects are too expensive and the money should be spent on education or healthcare. While these sectors are important, a country cannot function efficiently without a modern transport network. In conclusion, investing in public transport is not just a luxury, but a necessity for a more organized and healthy society.

## Essay B2-2. The advantages and disadvantages of working from home.

Since the pandemic, working from home has become a common practice for many companies around the world. This shift has brought both benefits and challenges for employees and employers alike. One of the primary advantages is the flexibility it offers. Employees can manage their time more effectively and avoid the daily stress of commuting through heavy traffic. This is particularly beneficial for parents who need to look after their children. Furthermore, companies can save money on office rent and electricity. On the other hand, there are several disadvantages. Many people find it difficult to separate their professional and personal lives, leading to "burnout." Additionally, the lack of face-to-face interaction can weaken the team spirit and slow down communication. In my view, a hybrid model is the best solution. This allows workers to enjoy the comfort of their homes for part of the week while still maintaining social connections at the office. To summarize, remote work is a great innovation, but it requires discipline and balance to be successful.

## Essay B2-3. Is it better to study alone or in a group?

University students often debate whether studying alone or in a group leads to better academic results. In my opinion, both methods have their merits, but studying alone is generally more effective for deep concentration. When you study by yourself, you can focus entirely on the topics that you find difficult without any distractions. You can set your own pace and spend more time on complex theories. For example, when I prepare for my English exams, I prefer to be in a quiet library where I can memorize vocabulary and practice grammar. However, group study can be very helpful for brainstorming and solving problems. If one student doesn't understand a concept, another student can explain it in a simple way. This encourages active learning and communication skills. Nevertheless, groups can sometimes become too social, and students may end up wasting time talking about other things. In conclusion, while group study is good for revision and sharing ideas, individual study remains the most reliable way to master a subject.

## Essay B2-4. The impact of technology on modern communication.

Technology has fundamentally changed the way humans interact with each other. In the past, people wrote letters or used landline phones, but today, social media and instant messaging are the dominant forms of communication. One major benefit is the speed of information. We can contact anyone in the world instantly, which has made the world feel like a small village. This is especially useful for international business and maintaining long-distance relationships. Moreover, technology has made communication more visual through video calls and photo sharing. However, many critics argue that technology has made our relationships more superficial. People often prefer to send a text message instead of having a real conversation, which can lead to misunderstandings. Furthermore, the constant use of smartphones has reduced our attention spans during social gatherings. In conclusion, while technology has made communication faster and more convenient, we must be careful not to lose the "human touch" that is essential for meaningful relationships.

## Essay B2-5. Should the voting age be lowered to sixteen?

In many countries, there is an ongoing debate about whether sixteen-year-olds should be allowed to vote in national elections. Supporters of this idea argue that young people are directly affected by government policies on education and the environment, so they should have a voice in the decision-making process. At sixteen, many teenagers are already working part-time and paying taxes, which means they are contributing to the economy. Furthermore, allowing them to vote could encourage early political engagement and increase voter turnout in the future. On the other hand, opponents claim that sixteen-year-olds lack the emotional maturity and life experience necessary to make informed political choices. They argue that teenagers might be easily influenced by social media trends or the opinions of their parents. In my opinion, sixteen is a bit too young for such a heavy responsibility. It is better to wait until eighteen when students have completed their basic education and have a more stable understanding of the world. In conclusion, while it is important to listen to the youth, the current voting age is appropriate.

## Essay B2-6. The environmental impact of tourism.

Tourism is a vital sector for many nations, providing jobs and boosting the economy. However, the environmental impact of mass tourism is a growing concern that cannot be ignored. On the one hand, tourism brings significant financial resources that can be used to protect natural landmarks and historical sites. For instance, the entrance fees for national parks in Egypt help fund the conservation of coral reefs in the Red Sea. On the other hand, the negative effects are numerous. Large hotels often consume massive amounts of water and electricity, which can lead to shortages for local residents. Furthermore, the increase in air travel contributes significantly to global carbon emissions. Waste management is another major issue, as tourists often leave plastic bottles and trash in pristine environments. In my opinion, we need to promote "sustainable tourism." This means encouraging visitors to respect nature and supporting businesses that use renewable energy. In conclusion, while tourism is economically beneficial, we must implement stricter environmental regulations to ensure that our natural treasures are preserved for future generations.

## Essay B2-7. Should animals be used for scientific research?

The use of animals in scientific research has been a controversial topic for decades. Some people believe that it is a necessary evil for medical progress, while others argue that it is a cruel and unethical practice. Proponents of animal testing point out that almost every major medical breakthrough, such as vaccines and antibiotics, was developed through animal research. They argue that testing new drugs on humans would be too dangerous without first checking their safety on animals. From this perspective, the life of a human is more valuable than that of a mouse or a rabbit. Conversely, animal rights activists argue that animals are sentient beings that feel pain and fear. They believe it is immoral to subject them to suffering for human benefit. Moreover, they point out that results from animal tests are not always applicable to humans because of biological differences. In my view, we should aim to reduce animal testing by investing in alternative methods, such as computer modeling and lab-grown human cells. In conclusion, while animal research has saved lives, we must strive for more humane and advanced scientific techniques.

## Essay B2-8. Are university exams a fair way to test knowledge?

In most educational systems, standardized exams are the primary method used to evaluate a student's performance. However, there is a debate about whether these exams truly reflect a person's intelligence and knowledge. One major advantage of exams is that they are objective. Every student takes the same test under the same conditions, which makes it easier for universities to compare results fairly. It also encourages students to review the entire syllabus and work hard under pressure. However, there are significant drawbacks. Exams often test memory rather than deep understanding. A student might memorize facts for a day and forget them immediately after the test. Additionally, some very talented students suffer from "exam anxiety," which prevents them from performing well regardless of their knowledge. In my opinion, a combination of exams and continuous assessment, such as projects and presentations, is a better approach. This provides a more complete picture of a student's abilities throughout the year. To summarize, while exams have their place in education, they should not be the only factor in determining a student's success.

## Essay B2-9. The challenges of young people moving abroad for work.

In recent years, an increasing number of young Egyptians have decided to move abroad to find better job opportunities in Europe or the Gulf. While this can lead to a more successful career, it also presents several challenges. The main benefit is obviously the higher salary and the chance to gain international experience. Working in a different country can broaden one's perspective and improve professional skills. However, the emotional cost is often high. Many young people suffer from homesickness and the difficulty of adapting to a new culture and language. Being away from family and friends can lead to feelings of isolation and loneliness. Furthermore, the "brain drain" phenomenon is a problem for the home country, as it loses its most talented and educated young citizens. In my view, moving abroad is a great opportunity for personal growth, but it requires a lot of courage and preparation. In conclusion, young people should weigh the financial gains against the emotional challenges before deciding to leave their homeland for a long time.

## Essay B2-10. The necessity of shifting to renewable energy.

As global warming continues to threaten our planet, the transition from fossil fuels to renewable energy sources has become a global priority. In countries like Egypt, where sunlight is abundant, solar energy offers a promising solution to our energy needs. The primary advantage of renewable energy is that it is clean and does not produce greenhouse gases. This is essential for reducing air pollution and slowing down climate change. Additionally, wind and solar power are infinite, unlike oil and gas, which will eventually run out. However, the initial cost of building solar plants and wind farms is very high, and some developing countries might struggle to afford the technology. There is also the issue of reliability, as these energy sources depend on the weather. Despite these challenges, I believe that the long-term benefits far outweigh the costs. Governments must provide incentives for green energy projects to protect the environment. In conclusion, shifting to renewable energy is not just an option but a necessity for the survival of our civilization and the health of the earth.

## Essay B2-11. How advertising influences our choices.

In the modern world, we are surrounded by advertisements on television, social media, and billboards in the streets. While some people believe that advertising provides useful information, I think it often manipulates our choices and encourages unnecessary consumption. On the positive side, adverts inform us about new products and special offers, which can save us money. For example, when a new smartphone is released, we can compare its features through different commercials. However, the primary goal of advertising is to persuade us that we "need" things that we actually don't. Companies use celebrities and beautiful images to make their products look perfect. This creates a lot of pressure, especially on young people, to buy expensive brands to feel successful or popular. Furthermore, many adverts for fast food and sugary drinks are aimed at children, which contributes to health problems. In my opinion, we should be more critical of what we see in the media. In conclusion, while advertising is a necessary part of the economy, we must learn to distinguish between our real needs and the desires created by marketing.

## Essay B2-12. The balance between career success and personal life.

For many people today, achieving professional success is a top priority, but this often comes at the expense of their personal lives and health. In my view, finding a balance between work and family is the most important factor for long-term happiness. On the one hand, a successful career provides financial stability and a sense of achievement. It allows us to afford a comfortable life and support our families. Many people in Egypt work two jobs to ensure their children get a good education. On the other hand, working too many hours can lead to chronic stress and a lack of time for social relationships. If you are always at the office, you will miss important moments with your children and friends. Moreover, stress can lead to serious health issues like high blood pressure. I believe that companies should encourage flexible working hours to help employees maintain their mental well-being. To summarize, although hard work is necessary for success, it should not be the only thing in our lives. We must make time for the people we love and for our own relaxation.

## Essay B2-13. Should animals be kept in zoos?

The existence of zoos is a controversial topic that raises important ethical questions about our relationship with nature. Some people argue that zoos are essential for education and conservation, while others believe that keeping animals in cages is cruel. Those who support zoos point out that they allow people to see exotic animals that they would never see in real life. This can inspire children to care more about the environment. Furthermore, many zoos have successful breeding programs for endangered species, which helps to prevent extinction. Conversely, critics argue that a zoo can never replicate an animal's natural habitat. Animals in captivity often show signs of stress and boredom because they cannot hunt or roam freely over long distances. In my opinion, we should only support zoos that prioritize animal welfare and research rather than entertainment. Large safari parks are a better alternative because they provide more space and a more natural environment. In conclusion, while zoos play a role in education, we must ensure that the animals are treated with the respect and dignity they deserve.

## Essay B2-14. The impact of urbanization on local communities.

Urbanization is the process of people moving from rural areas to cities in search of better opportunities. While this movement can drive economic development, it also creates significant challenges for local communities. One major advantage of urbanization is the concentration of services. In big cities, people have better access to advanced healthcare, diverse education, and a wider variety of jobs. This is why many young people leave their villages to settle in Cairo or Alexandria. However, rapid urbanization often leads to overpopulation and the growth of informal settlements or "slums." These areas often lack basic services like clean water and electricity. Furthermore, the traditional social bonds that exist in small villages are often lost in the city, where people tend to live more isolated lives. In my view, the government should invest more in rural areas to create jobs there and reduce the pressure on big cities. In conclusion, urbanization is an inevitable part of development, but it must be managed carefully to avoid social and environmental problems.

## Essay B2-15. The importance of celebrating cultural festivals.

Cultural festivals are an essential part of a nation's identity and heritage. In Egypt, we have many beautiful festivals like Sham El-Nessim and Eid, which bring families and communities together. I believe that these celebrations are vital for maintaining our history and social harmony. Firstly, festivals allow us to pass down traditions to the younger generation. When children participate in these events, they learn about their ancestors and the values of their culture. Secondly, they promote social cohesion. During a festival, people often forget their differences and share food and joy with their neighbors. This strengthens the "social fabric" of the country. Moreover, festivals are a great way to attract tourists, who are interested in experiencing the authentic traditions of a place. This provides an economic boost to local businesses and artisans. However, we should be careful not to make these festivals too commercialized. In conclusion, cultural festivals are more than just holidays; they are a celebration of who we are, and we should protect them as part of our national treasure.

## Essay B2-16. The ethical debate over capital punishment.

Capital punishment, or the death penalty, remains one of the most controversial issues in modern legal systems. While many countries have abolished it, others continue to use it for the most serious crimes. Proponents of capital punishment argue that it serves as a powerful deterrent. If a criminal knows they might face death, they might think twice before committing a violent act. Furthermore, they believe it provides "retributive justice" for the victims' families, giving them a sense of closure. On the other hand, opponents argue that the death penalty is a violation of the fundamental right to life. They claim that it is inhumane and that a civilized society should not use violence to punish violence. A major concern is the risk of executing an innocent person, as legal systems are not perfect and mistakes can happen. Once a person is executed, the mistake cannot be corrected. In my opinion, life imprisonment without parole is a better alternative. It protects society while allowing for the possibility of correcting a wrong conviction. In conclusion, the death penalty is an outdated practice that should be replaced by more humane forms of justice.

## Essay B2-17. The impact of social media on self-esteem.

The rise of social media platforms like Instagram and TikTok has significantly altered how young people perceive themselves. While these platforms offer a way to connect with others, they also have a profound impact on self-esteem. One of the main issues is the "culture of comparison." People usually post only the best moments of their lives, often using filters to look perfect. When teenagers see these idealized images, they often feel that their own lives and bodies are not good enough. This can lead to feelings of inadequacy, anxiety, and even depression. Moreover, the "like" system creates a dependency on external validation. Many young people measure their self-worth by the number of likes or comments they receive on a photo. If a post doesn't perform well, they might feel rejected or unpopular. On the positive side, social media can be a source of inspiration and a place to find supportive communities. For example, groups that promote "body positivity" can help people feel more confident. In my view, we need to educate the youth about "digital literacy" so they understand that what they see online is often not reality. To summarize, social media is a powerful tool that can harm self-esteem if used without a critical mind.

## Essay B2-18. The pros and cons of mandatory military service.

In some countries, including Egypt, military service is mandatory for young men after they finish their education. This policy has both supporters and critics. Supporters argue that military service teaches young people discipline, responsibility, and teamwork. It prepares them for the challenges of adult life and gives them a sense of national pride. Furthermore, it ensures that the country has a large and ready force to defend its borders in case of a national emergency. During their service, many young men also learn technical skills that can be useful in their future careers. On the other hand, critics believe that mandatory service is an infringement on personal freedom. They argue that young people should have the right to choose their own path, whether it is starting a job or continuing their studies. Many believe that a professional, voluntary army is more effective than one made up of conscripts who might not want to be there. Additionally, spending one or two years in the military can delay a person's entry into the labor market, which can affect the economy. In my opinion, while the military is important, the duration of service should be minimized. In conclusion, mandatory service is a complex tradition that balances national security with individual rights.

## Essay B2-19. Plastic pollution and its threat to the oceans.

Plastic pollution has become one of the most urgent environmental crises of our time. Every year, millions of tons of plastic waste end up in the oceans, causing irreparable damage to marine ecosystems. One of the primary reasons for this is the widespread use of "single-use" plastics, such as straws, bags, and water bottles. These items are used for a few minutes but take hundreds of years to decompose. Marine animals, such as turtles and whales, often mistake plastic for food, which leads to injury or death. Furthermore, plastic breaks down into "microplastics," which are swallowed by fish and eventually enter the human food chain. This poses a potential risk to human health that we are only beginning to understand. In my view, the solution requires a combination of government action and individual responsibility. Governments should ban non-essential plastic items and invest in better recycling infrastructure. Meanwhile, individuals can reduce their plastic footprint by using reusable bags and bottles. In conclusion, the health of our oceans is vital for the survival of the planet, and we must take immediate action to end our addiction to plastic.

## Essay B2-20. The role of the arts in a STEM-focused world.

In recent years, there has been a strong emphasis on STEM subjects (Science, Technology, Engineering, and Mathematics) in the education system. While these fields are essential for economic growth, I believe that the arts still play a vital role in society. Firstly, the arts—including music, literature, and painting—foster creativity and innovation. Many great scientists and engineers are also musicians or artists because the arts teach us to think "outside the box" and see the world from different perspectives. Secondly, the arts are essential for emotional and social development. They allow us to express complex feelings and understand the experiences of others, which builds empathy. For example, reading a novel from a different culture can teach us more about humanity than a textbook. Moreover, the creative industries contribute significantly to the economy through tourism, film, and design. In my opinion, a well-rounded education should include both science and the arts. We should not view them as opposites but as complementary ways of understanding the human experience. In conclusion, even in a world dominated by technology, the arts remain the soul of our civilization.

## Essay B2-21. The impact of tourism on local culture.

Tourism is one of the main sources of income for many countries, especially Egypt. While it brings significant economic benefits, it also has a profound impact on local culture. On the positive side, tourism encourages the preservation of historical sites and traditional crafts. When tourists show interest in local culture, the community feels proud of its heritage and works hard to maintain it. For instance, the traditional markets in Luxor and Aswan have stayed the same for years because tourists love the authentic atmosphere. On the other hand, mass tourism can lead to "cultural dilution." This happens when local businesses change their traditions to please foreign visitors. For example, some restaurants might stop serving traditional food and offer only international dishes. Furthermore, the behavior of some tourists may not align with local values, which can lead to misunderstandings or social tension. In my opinion, we should promote cultural tourism that respects local traditions. In conclusion, tourism is a great way to share our culture with the world, but we must ensure that our unique identity is not lost in the process.

## Essay B2-22. Is social media replacing face-to-face communication?

In the last decade, social media platforms have become the primary way for people to stay connected. However, there is a growing debate about whether these digital tools are replacing real-life, face-to-face communication. In my view, while social media makes communication easier, it cannot replace the depth of physical interaction. One advantage of social media is that it allows us to talk to people instantly regardless of the distance. It is a great tool for maintaining long-distance relationships and sharing news quickly. However, digital communication lacks "non-verbal cues" such as body language and tone of voice. This can often lead to misunderstandings and a lack of emotional connection. Many people now prefer to send a text message instead of visiting a friend, which can lead to feelings of loneliness in the long run. Furthermore, when people are together, they often spend time looking at their phones instead of talking to each other. In conclusion, social media should be a supplement to our social lives, not a replacement for the meaningful experience of talking to someone in person.

## Essay B2-23. The benefits of learning music at an early age.

Many parents wonder whether they should encourage their children to learn a musical instrument. I believe that music education offers numerous cognitive and emotional benefits that are essential for a child's development. Firstly, learning music improves brain function. Studies have shown that children who play instruments like the piano or violin have better memory and mathematical skills. This is because reading music and coordinating hand movements require complex brain activity. Secondly, music is a great way for children to express their emotions. It can be a source of comfort and a way to reduce stress from school work. Moreover, practicing an instrument teaches children discipline and patience. It takes months of hard work to master a song, and this teaches them that success comes from persistence. On the other hand, some people argue that music is a distraction from academic subjects like science. In my opinion, music actually helps students perform better in all subjects. In conclusion, music is a powerful tool for personal growth, and every child should have the opportunity to explore it.

## Essay B2-24. Should school days be shorter?

The length of the school day is a topic of frequent discussion among educators and parents. Some argue that students spend too much time in the classroom, while others believe that more hours are necessary for a comprehensive education. In my opinion, shortening the school day would be beneficial for both students and teachers. One major benefit of shorter days is that students would have more time for extracurricular activities and rest. Currently, many students in Egypt leave home early and return late, leaving them with very little time for hobbies or physical exercise. This constant pressure can lead to burnout and a lack of interest in learning. Secondly, a shorter day would encourage "quality over quantity." If the hours were reduced, teachers would have to be more efficient and focus on the most important topics. However, critics argue that shorter days would be difficult for working parents who rely on the school to look after their children. While this is a valid point, I believe that a more balanced schedule would improve the overall mental health of the youth. In conclusion, a shorter school day would lead to a more relaxed and effective learning environment.

## Essay B2-25. How technology affects the fashion industry.

Technology has revolutionized almost every aspect of the fashion industry, from the way clothes are designed to how they are sold. In my view, these changes have made fashion more accessible but also raised some ethical concerns. One of the most significant changes is the rise of "e-commerce." Today, we can buy clothes from anywhere in the world using our smartphones. This has given smaller designers a chance to reach a global audience without needing a physical shop. Additionally, new technologies like 3D printing and smart fabrics are allowing for more creative and functional designs. However, technology has also led to the rise of "fast fashion." Because of automated factories, companies can produce thousands of new items every week at a very low cost. This encourages people to buy more clothes and throw them away quickly, which is devastating for the environment. Furthermore, the use of AI to predict trends can lead to a lack of originality in fashion. In conclusion, while technology has made the fashion world more dynamic and convenient, we must focus on using it to create a more sustainable and ethical industry.

## Essay B2-26. The impact of influencers on shopping habits.

In the age of social media, "influencers" have become a powerful force in the retail industry. Many companies now prefer to pay popular Instagram or YouTube stars to promote their products rather than using traditional advertising. In my opinion, this trend has a significant impact on how young people spend their money, and not always in a positive way. One advantage is that influencers often provide honest reviews and show how a product looks in real life. This can help consumers make more informed choices. However, the problem is that many followers feel a "parasocial relationship" with these influencers, meaning they trust them like a friend. This makes them more likely to buy things they don't need just to imitate the influencer's lifestyle. Furthermore, this leads to a culture of materialism, where self-worth is tied to owning the latest trends. In conclusion, while influencers can be a helpful source of information, we must remain critical and avoid being manipulated by the perfect images we see online.

## Essay B2-27. Urban greening vs. industrial development.

As cities in Egypt continue to expand, there is a constant conflict between the need for industrial development and the preservation of green spaces. While factories and infrastructure are essential for economic growth, I believe that urban greening should be a priority for the health of the population. Firstly, industrial development often leads to severe air and noise pollution, which can cause long-term health problems for city dwellers. Green spaces, such as parks and "urban forests," act as filters that clean the air and provide a quiet place for relaxation. Secondly, the presence of nature in a city has a positive effect on mental well-being, reducing stress and anxiety. However, critics argue that the country needs more factories to create jobs and reduce poverty. While this is true, development should be sustainable. We can build industrial zones outside the residential areas and ensure that every new neighborhood has a minimum amount of green space. In conclusion, a balance between industry and nature is necessary for a healthy and prosperous urban life.

## Essay B2-28. The future of printed books in a digital world.

With the invention of e-readers and tablets, many people predicted that printed books would eventually disappear. However, despite the convenience of digital reading, printed books remain popular among readers of all ages. In my view, both formats have a place in our lives. The main advantage of digital books is their portability; you can carry a whole library in one small device, which is perfect for traveling or commuting. They are also often cheaper than physical books. On the other hand, many readers still prefer the "tactile experience" of holding a real book and turning its pages. For many, reading a physical book provides a break from the screens that dominate our work lives. Additionally, a bookshelf full of books is a source of pride and a part of a home's identity. In my opinion, technology will not kill the printed book, but rather change how we use it. We might use digital books for quick information and printed books for deep reading. In conclusion, the medium may change, but the love for reading is permanent.

## Essay B2-29. Is competition healthy for students?

The role of competition in education is a subject of much debate among teachers and psychologists. Some believe that competing for grades and prizes motivates students, while others worry about the negative impact on their mental health. In my opinion, healthy competition can be beneficial, but it should not be the only focus of the school system. On the positive side, competition prepares students for the "real world," where they will have to compete for jobs and promotions. it encourages them to work harder and strive for excellence. For example, sports competitions teach students about teamwork and how to handle both victory and defeat. However, excessive pressure to be the "best" can lead to high levels of anxiety and a loss of interest in the subject itself. It can also discourage students who are not naturally competitive. I believe that schools should also emphasize cooperation and personal growth. In conclusion, competition is a good tool for motivation as long as it is balanced with a supportive and collaborative environment.

## Essay B2-30. Remote work and the potential for global collaboration.

The shift toward remote work has opened up new possibilities for collaboration between people from different countries and cultures. Thanks to digital tools like Zoom and Slack, a team can now consist of individuals living in Cairo, London, and Tokyo. I believe that this trend will lead to a more innovative and inclusive global economy. Firstly, remote work allows companies to hire the most talented people regardless of their location. This gives people in developing countries the chance to work for international corporations without leaving their families. Secondly, working in a diverse, global team brings different perspectives and ideas, which can lead to more creative solutions to problems. However, there are challenges, such as managing different time zones and cultural misunderstandings in communication. Additionally, the lack of physical interaction can make it difficult to build trust among team members. In my view, the benefits of global collaboration far outweigh these obstacles. In conclusion, remote work is not just a temporary change but a fundamental shift that will make our professional world more connected and diverse.

## Essay B2-31. The qualities of a successful entrepreneur.

In recent years, many young people in Egypt have become interested in starting their own businesses instead of looking for traditional office jobs. To be a successful entrepreneur, I believe that several key qualities are necessary. Firstly, resilience is essential. Starting a business is full of challenges and many startups fail in the first year. An entrepreneur must be able to handle failure and keep going without losing motivation. Secondly, creativity is vital for finding new solutions to old problems. In a competitive market, you need to offer something unique to attract customers. Furthermore, good communication skills are important for leading a team and persuading investors to support your ideas. On the other hand, some people argue that having a lot of money is the most important factor. While capital is necessary, many successful companies started with a very small budget. In my opinion, the person's mindset is more important than their bank account. In conclusion, being an entrepreneur is difficult, but with hard work, creativity, and resilience, anyone can achieve their dreams.

## Essay B2-32. The pros and cons of nuclear energy.

As the world faces a growing energy crisis, many countries are reconsidering nuclear energy as a solution. This source of power has both significant advantages and serious risks. One of the primary benefits is that nuclear power plants produce a massive amount of electricity without emitting greenhouse gases. This makes it a much cleaner alternative to coal or oil. Additionally, nuclear energy is more reliable than solar or wind power because it does not depend on the weather. However, the disadvantages are very concerning. The most serious risk is a nuclear accident, which can have devastating effects on human health and the environment for thousands of years. Furthermore, the problem of how to safely store radioactive waste has not been fully solved. In my view, while nuclear energy can help reduce global warming, we should prioritize investing in safer renewable sources first. To summarize, nuclear power is a powerful tool for modern society, but the potential for catastrophe means it must be used with extreme caution.

## Essay B2-33. Should school children be allowed to use tablets instead of books?

The digital revolution has entered the classroom, and many schools are now replacing traditional textbooks with tablets. I believe that this change has more benefits than drawbacks for the modern student. One major advantage of tablets is the "weight factor." Instead of carrying a heavy backpack full of books, which can cause back pain, a student only needs one small device. This is much more convenient and healthy. Moreover, tablets offer interactive learning experiences. Students can watch videos, use educational apps, and search for information instantly. This makes the lessons more engaging and fun. On the other hand, some parents worry that tablets can be a source of distraction, as students might play games or use social media during class. Additionally, looking at a screen for many hours can be bad for the eyes. In my opinion, these problems can be solved with proper supervision and by using high-quality screens. In conclusion, tablets are a modern tool that can significantly improve the quality of education if used responsibly.

## Essay B2-34. The importance of preserving local dialects.

Language is more than just a tool for communication; it is a vital part of a person's identity and culture. In many parts of the world, local dialects are disappearing as people move to cities and use more standardized versions of the language. I believe that preserving these dialects is essential for maintaining our cultural diversity. Firstly, a dialect often contains unique words and expressions that reflect the history and environment of a specific region. If a dialect dies, that local knowledge is lost forever. Secondly, speaking a dialect gives people a sense of belonging to their community. In Egypt, for example, the dialects of Alexandria, Upper Egypt, and the Delta are all different and beautiful in their own way. However, some argue that everyone should speak the same way to make communication easier. While a standard language is useful for business and education, we should still encourage people to use their dialects at home and in their communities. In conclusion, local dialects are a national treasure, and we should be proud of our linguistic variety.

## Essay B2-35. Is the traditional family dinner disappearing?

In the past, the family dinner was a sacred time when everyone sat together to share food and talk about their day. Today, because of busy schedules and the influence of technology, many people believe that this tradition is disappearing. In my view, this is a negative trend that could affect family relationships. One reason for this change is that parents and children often have different schedules. Many people work late, and students have many extra lessons, so they eat at different times. Furthermore, even when they eat together, many people look at their phones instead of talking to each other. This leads to a lack of communication and a feeling of isolation within the family. In my opinion, families should make a conscious effort to have dinner together at least a few times a week without any electronic devices. This is the best way to strengthen bonds and support each other. In conclusion, while our lives have become more hectic, we should not lose the simple but important tradition of the family meal.

## Essay B2-36. Should museums be free for everyone?

Museums are the guardians of a nation's history and culture, yet there is a constant debate about whether they should charge an entrance fee. In my opinion, making museums free for everyone would have significant benefits for education and society. Firstly, free admission would encourage more people to learn about their heritage. Many low-income families in Egypt cannot afford the tickets for places like the Grand Egyptian Museum, which means their children miss out on vital educational experiences. If it were free, everyone would have equal access to knowledge regardless of their financial status. Secondly, free museums attract more international tourists, who would then spend money in local shops and restaurants, boosting the economy indirectly. On the other hand, some argue that museums need the revenue from tickets to pay for maintenance, security, and the preservation of artifacts. While this is a valid point, the government could fund museums through taxes, viewing them as a public service similar to libraries. In conclusion, the cultural and educational value of free museums far outweighs the cost of admission.

## Essay B2-37. The decline of traditional letter writing.

Before the age of emails and instant messaging, writing letters was the primary way for people to stay in touch over long distances. Today, this tradition has almost disappeared, which I believe is a loss for our personal connections. The main reason for the decline is clearly the speed and convenience of modern technology. Sending a WhatsApp message takes seconds, while a letter takes days to arrive. However, a handwritten letter has a "personal touch" that an email lacks. When you receive a letter, you know that the person spent time and effort to think about you and write to you. It is something physical that you can keep for years and read again to remember a specific moment in your life. Digital messages, on the other hand, are often deleted or forgotten in a sea of notifications. In my opinion, we should try to revive this tradition for special occasions like birthdays or anniversaries. In conclusion, while technology has made communication more efficient, it has also made it less meaningful, and we should not let the art of letter writing die completely.

## Essay B2-38. The impact of celebrity culture on modern youth.

In the digital age, celebrities have more influence on young people than ever before, thanks to social media platforms like Instagram and TikTok. While some celebrities use their fame for good, I believe that the overall impact of celebrity culture on the youth is often negative. One major problem is the promotion of unrealistic lifestyles. Many stars post photos of their expensive cars, designer clothes, and luxury vacations, which can make teenagers feel dissatisfied with their own lives. This leads to a culture of materialism where "having" is more important than "being." Furthermore, many celebrities promote unhealthy beauty standards through filtered images, contributing to body image issues among young girls. On the positive side, some celebrities use their platform to raise awareness about climate change or mental health, providing positive role models for their fans. However, these are often the minority. In my view, parents and teachers should help young people understand that fame does not always equal success. In conclusion, celebrity culture is a powerful force that can distort a young person's values if they are not careful.

## Essay B2-39. The role of sports in international diplomacy.

Sports have always been more than just physical competition; they are a powerful tool for bringing nations together. I believe that international sporting events, like the World Cup or the Olympics, play a vital role in global diplomacy and peace. Firstly, sports provide a platform for people from different cultures to meet and interact in a friendly environment. When fans from different countries cheer together in a stadium, they forget their political differences and focus on their shared passion. This helps to break down stereotypes and build mutual respect. Secondly, "sports diplomacy" can sometimes open doors for political dialogue. A famous example is the "Ping-Pong Diplomacy" between the USA and China in the 1970s, which helped improve their relationship. However, sports can also be used for nationalistic purposes or to cover up human rights issues, a practice known as "sportswashing." Despite these risks, I believe the positive potential of sports is much greater. In conclusion, sports are a universal language that can foster understanding and cooperation in a fragmented world.

## Essay B2-40. The advantages of a cashless society.

In many developed countries, the use of physical cash is rapidly declining as people switch to digital payments and credit cards. While some are worried about this shift, I believe that moving toward a cashless society offers several significant advantages. The primary benefit is convenience; you no longer need to carry a heavy wallet or look for an ATM to withdraw money. Payments can be made instantly using a smartphone or a watch, which speeds up transactions in shops and restaurants. Furthermore, a cashless system is much more secure and transparent. It is much harder to hide illegal activities, such as tax evasion or money laundering, when every transaction is recorded digitally. Additionally, losing your phone is safer than losing a bag of cash, as you can easily block your accounts. However, a major disadvantage is the "digital divide," as elderly people or those in rural areas might struggle to use the technology. In my opinion, the government should ensure that everyone has access to digital tools. In conclusion, a cashless society is an inevitable and positive step toward a more efficient and secure economy.

## Essay B2-41. The impact of remote learning on student discipline.

The rapid transition to online education has brought many conveniences, but it has also raised concerns about student discipline and motivation. In my opinion, while remote learning offers flexibility, it requires a much higher level of self-discipline than traditional classroom learning. One major challenge is the lack of direct supervision. In a physical classroom, the presence of the teacher and other students creates a structured environment that encourages focus. At home, however, students are surrounded by distractions, such as social media, family members, and the comfort of their own beds. This can easily lead to procrastination and a lack of effort. Furthermore, the absence of a fixed schedule can make students lazy and unorganized. On the positive side, online learning teaches students how to manage their own time and be independent learners. These are essential skills for university and professional life. However, many students in Egypt struggle with this responsibility. In conclusion, for remote learning to be successful, students must develop strong time-management skills and stay motivated without constant supervision from teachers.

## Essay B2-42. Is the gap between the rich and the poor widening?

In many societies today, there is a growing concern that the economic gap between the wealthy and the impoverished is increasing. I believe that this inequality is a serious problem that could lead to social instability. One reason for this widening gap is the difference in educational opportunities. Rich families can afford private schools and international universities, which give their children better job prospects and higher salaries. In contrast, children from poor backgrounds often have access to lower-quality education, which limits their future income. Furthermore, the rise of technology and automation has replaced many low-skilled jobs, making it harder for the poor to find stable work. On the other hand, some argue that the "middle class" is growing in many developing countries, providing more opportunities for everyone. While this may be true in some regions, the concentration of wealth in the hands of a small percentage of the population remains a global issue. In my opinion, governments should implement fairer tax systems and invest more in public services. In conclusion, reducing economic inequality is essential for creating a more just and peaceful world.

## Essay B2-43. The ethics of genetic engineering in agriculture.

Genetic engineering has allowed scientists to create crops that are resistant to pests and can grow in harsh climates. While this technology could solve the problem of global hunger, it also raises several ethical and environmental concerns. One of the main advantages of genetically modified (GM) crops is their high yield. In countries like Egypt, where water is scarce and the population is growing, these crops could ensure food security for everyone. Furthermore, they reduce the need for chemical pesticides, which is better for the health of consumers. However, critics argue that we do not yet know the long-term effects of GM food on human health. There is also the risk of "genetic pollution," where modified genes could spread to wild plants and disrupt the local ecosystem. Moreover, a few large corporations control the technology, which can make small farmers dependent on them for seeds. In my view, we should use genetic engineering carefully and with strict regulations. In conclusion, while GM technology offers great potential, we must prioritize safety and environmental protection over corporate profit.

## Essay B2-44. Should the government subsidize electric cars?

As air pollution and climate change become more urgent issues, many people are calling for the government to provide financial support for the purchase of electric cars. In my opinion, subsidizing electric vehicles (EVs) is a necessary step toward a greener future. Firstly, EVs produce zero emissions, which would significantly improve the air quality in crowded cities like Cairo. This would reduce respiratory diseases and healthcare costs in the long run. Secondly, by encouraging people to switch to electric cars, the government can reduce the country's dependence on imported oil and gas. This would make the economy more stable and resilient. However, critics argue that subsidies are a waste of taxpayers' money because electric cars are still very expensive and only the rich can afford them. They believe the money should be spent on public transport instead. While public transport is important, I believe that both sectors need investment. In conclusion, providing incentives for electric cars is a smart environmental and economic policy that will benefit everyone in the future.

## Essay B2-45. The role of traditional media in a digital age.

With the rise of social media and citizen journalism, many people have started to question the relevance of traditional media, such as newspapers and television news. I believe that while the format of media is changing, the role of professional journalism is more important than ever. The primary advantage of traditional media is its reliability. Professional journalists are trained to check their facts and follow ethical guidelines before publishing a story. In an age of "fake news" and misinformation on social media, we need trusted sources that provide accurate information. Furthermore, traditional media outlets often have the resources to conduct deep investigative journalism that uncovers corruption and holds the powerful accountable. On the other hand, social media is much faster and allows for more diverse voices to be heard. However, the lack of editorial oversight means that anyone can spread falsehoods. In my view, we should use social media for quick updates but rely on traditional media for a deeper understanding of the news. In conclusion, professional journalism remains a pillar of democracy and truth in our digital world.

## Essay B2-46. The growth of space tourism: A luxury or a necessity?

In recent years, private companies have started offering space travel to wealthy individuals. While some see this as a great leap for humanity, others view it as an unnecessary luxury for the rich. In my opinion, while space tourism is an impressive achievement, we should focus our resources on more urgent problems on Earth. One advantage of space tourism is that it could fund further scientific research and help us develop new technologies. For example, the innovations needed for space travel could lead to more efficient energy sources or better materials for construction. On the other hand, the environmental impact is a major concern. Rocket launches consume enormous amounts of fuel and release carbon dioxide into the atmosphere, contributing to global warming. Furthermore, it seems unfair to spend billions of dollars on a few minutes of "fun" for billionaires while millions of people still lack basic needs like food and medicine. In conclusion, space exploration is important, but tourism in space should not be a priority until we solve our environmental and social crises on Earth.

## Essay B2-47. The impact of fast fashion on the environment.

The fashion industry has changed dramatically with the rise of "fast fashion" brands that produce cheap, trendy clothes at an incredible speed. While this allows people to look stylish for a low price, I believe that the environmental and ethical costs are far too high. Firstly, the production of these clothes uses a massive amount of water and toxic chemicals, which pollute the rivers in many developing countries. Furthermore, since the clothes are made from low-quality materials, they are often thrown away after only a few months, leading to a huge increase in landfill waste. Secondly, there are serious concerns about the working conditions in the factories where these clothes are made. Many workers are paid very low wages and work in dangerous environments. In my view, consumers should switch to "slow fashion" by buying fewer but higher-quality items that last longer. Governments should also implement stricter regulations on the textile industry. In conclusion, fast fashion is a major threat to our planet, and we must change our shopping habits to protect the environment.

## Essay B2-48. The potential of renewable energy in Egypt.

Egypt is a country with vast natural resources that could make it a leader in the renewable energy sector. I believe that shifting toward solar and wind power is essential for our economic and environmental future. Firstly, Egypt has some of the highest levels of solar radiation in the world. Projects like the Benban Solar Park show that we can generate a significant portion of our electricity from the sun. This would reduce our dependence on natural gas and oil, which are finite and contribute to pollution. Secondly, the coastal areas along the Red Sea provide excellent conditions for wind farms. These projects could create thousands of new jobs for young engineers and workers. However, the high cost of the initial technology and the need for a modern "smart grid" are significant obstacles. Despite these challenges, the long-term benefits of clean, cheap energy are undeniable. In conclusion, the government should continue to invest in renewable energy to ensure a sustainable and prosperous future for the next generation of Egyptians.

## Essay B2-49. Should the government censor the internet?

The question of whether the government should have the power to censor internet content is a highly debated topic. While some argue that it is necessary for national security, I believe that free access to information is a fundamental right that should be protected. On the one hand, limited censorship could be used to stop the spread of illegal activities, such as terrorism or child exploitation. It can also help prevent "hate speech" that might lead to violence in society. On the other hand, there is a risk that governments will use censorship to silence political opponents and hide the truth from the public. This can stifle creativity and prevent citizens from having an informed debate about important issues. Furthermore, in a globalized world, it is very difficult to block information effectively, as people will always find ways to bypass the filters. In my opinion, education and digital literacy are better solutions than censorship. In conclusion, while we must protect people from harm, the internet should remain an open platform for the free exchange of ideas.

## Essay B2-50. Working in a team vs. working alone.

In most professional environments, employees are often required to work in teams to achieve specific goals. While some people prefer the independence of working alone, I believe that teamwork is generally more effective and rewarding. One major benefit of working in a team is the "pooling of ideas." Different people bring different perspectives and skills to a project, which often leads to more creative solutions. For example, in a marketing team, one person might be good at design while another is an expert in data analysis. Secondly, working together allows for a more efficient distribution of the workload, reducing the stress on a single person. However, teamwork can sometimes lead to conflicts or "social loafing," where some members don't work as hard as others. Working alone, conversely, allows for total concentration and complete control over the final result. In my view, the best approach depends on the task, but for complex projects, a team is always better. In conclusion, teamwork fosters innovation and builds strong professional relationships that are essential for a successful career.

# Level C1 (Advanced)

## Essay C1-1. The tension between globalization and cultural identity.

The phenomenon of globalization has undoubtedly fostered unprecedented levels of economic interdependence and cross-cultural exchange. However, this global integration has simultaneously sparked a contentious debate regarding the potential erosion of unique cultural identities. Critics argue that the "McDonaldization" of society leads to a homogenous global culture, where local traditions and languages are marginalized by dominant Western paradigms. In the Egyptian context, for instance, there is a palpable concern among intellectuals that the youth are increasingly gravitating towards globalized digital content, often at the expense of their linguistic heritage and traditional values. Nevertheless, it is reductive to view globalization as a purely destructive force. When managed strategically, it can provide a platform for cultural synthesis and the global promotion of local arts. The key lies in "glocalization"—the ability to adapt global influences to fit local contexts without losing one's cultural essence. Ultimately, preserving cultural identity in a globalized world requires a proactive educational approach that emphasizes both global citizenship and deep-rooted local awareness.

## Essay C1-2. Reassessing the role of meritocracy in contemporary labor markets.

The concept of meritocracy—a system where advancement is based solely on individual ability and effort—is often championed as the pinnacle of social justice. In theory, it ensures that the most competent individuals occupy the most influential roles, thereby maximizing societal efficiency. However, a closer examination reveals that the "playing field" is rarely level. Socio-economic background, access to elite education, and professional networking often play a more decisive role than innate talent. In many developing economies, the myth of meritocracy can inadvertently justify systemic inequalities, as those at the top are seen as having "earned" their status, while the systemic barriers facing the underprivileged are ignored. To truly achieve a meritocratic society, structural reforms are necessary to equalize opportunity from early childhood. This includes universal access to high-quality education and the elimination of nepotism in recruitment processes. Without these interventions, meritocracy remains a hollow ideal that obscures the persistence of inherited privilege. Therefore, we must move beyond the rhetoric of merit and focus on the practicalities of equity and social mobility.

## Essay C1-3. Ethical implications of Artificial Intelligence in judicial systems.

The implementation of Artificial Intelligence (AI) within the judiciary is no longer a futuristic concept but a burgeoning reality. Proponents argue that algorithmic decision-making can eliminate human bias, ensuring more consistent and objective legal outcomes. AI can analyze vast datasets of legal precedents far more efficiently than any human clerk, potentially reducing the massive backlogs that plague many legal systems. However, the ethical risks are profound. AI models are trained on historical data, which often reflects past societal biases; consequently, there is a significant risk that algorithms will perpetuate and even amplify systemic discrimination. Furthermore, the "black box" nature of complex AI systems poses a challenge to the principle of transparency. A defendant has a fundamental right to understand the reasoning behind a judicial decision, a requirement that purely mathematical models struggle to satisfy. While AI can certainly serve as a valuable diagnostic tool for legal research, the final moral and legal judgment must remain a human prerogative. The goal should be to augment human expertise with technological precision, rather than abdicating our judicial responsibilities to unfeeling code.

## Essay C1-4. The necessity of sustainable urban planning in the 21st century.

As the global population becomes increasingly urbanized, the traditional models of city development are proving to be ecologically and socially unsustainable. Modern urban planning must transition toward a paradigm that prioritizes environmental resilience and social inclusivity. This shift involves moving away from car-centric infrastructures toward "15-minute cities," where essential services are accessible via walking or cycling. Such a transition not only reduces carbon emissions but also revitalizes the social fabric of neighborhoods by encouraging spontaneous human interaction. In rapidly expanding metropolises, the integration of green spaces is equally vital; these "urban lungs" mitigate the heat-island effect and provide psychological benefits to residents. However, the primary obstacle to sustainable planning is often political and economic inertia. Short-term profits from real estate speculation frequently override long-term ecological considerations. To overcome this, governments must implement rigorous zoning laws and provide incentives for green building technologies. Sustainable urbanism is not merely an aesthetic choice; it is a critical strategy for ensuring that our cities remain habitable in an era of accelerating climate change.

## Essay C1-5. The crisis of misinformation in the digital age.

The democratization of information facilitated by the internet has, paradoxically, led to a crisis of truth. The rapid proliferation of misinformation and "fake news" poses a significant threat to democratic stability and public health. Unlike traditional media, which—ideally—operates under editorial oversight and fact-checking protocols, digital platforms are designed to maximize engagement, often through the promotion of sensationalist or polarizing content. This creates "echo chambers" where individuals are only exposed to information that confirms their existing biases, making them more susceptible to manipulation. Addressing this crisis requires a multi-faceted approach. While technological solutions, such as AI-driven fact-checking, are helpful, they are not a panacea. The most effective defense against misinformation is the cultivation of critical media literacy. Education systems must evolve to teach students how to evaluate sources, recognize logical fallacies, and understand the economic incentives behind digital content. Furthermore, social media corporations must be held accountable for the algorithms that facilitate the spread of harmful falsehoods. In the absence of such measures, the digital public square risks becoming a source of fragmentation rather than enlightenment.

## Essay C1-6. The erosion of privacy in the age of surveillance capitalism.

The pervasive nature of digital surveillance has fundamentally altered the relationship between the individual and the state, as well as the corporate world. In what has been termed "surveillance capitalism," our personal data has become a commodity, harvested by tech giants to predict and manipulate consumer behavior. While proponents of these technologies highlight the convenience of personalized services and enhanced national security, the cost to individual privacy is staggering. The constant tracking of our digital footprints—ranging from location data to private correspondences—creates a "panopticon" effect, where the mere awareness of being watched can stifle freedom of expression and dissent. In many societies, this data is not merely used for marketing but is integrated into social credit systems or predictive policing, raising profound questions about agency and autonomy. Furthermore, the lack of robust international regulations allows for the exploitation of data without meaningful consent. To safeguard the democratic ideal of privacy, we must move beyond simple "terms and conditions" and demand structural transparency and data sovereignty. Privacy is not a luxury to be traded for convenience; it is a fundamental human right that underpins the very possibility of a free society.

## Essay C1-7. Universal Basic Income: A solution to technological unemployment?

The prospect of widespread automation and the rise of Artificial Intelligence have reignited the debate over Universal Basic Income (UBI)—a policy where every citizen receives a guaranteed, unconditional sum of money from the government. Critics often dismiss UBI as an idealistic fantasy that would disincentivize work and bankrupt the state. However, as AI begins to displace not only manual labor but also high-skilled cognitive roles, the traditional link between labor and survival is becoming increasingly tenuous. UBI offers a potential safety net that could prevent a socioeconomic crisis characterized by extreme inequality and social unrest. Beyond mere survival, UBI could empower individuals to pursue creative, entrepreneurial, or caregiving roles that are currently undervalued by the market. This shift could lead to a more human-centric economy, where work is a choice rather than a desperate necessity. Nevertheless, the fiscal feasibility of such a program remains a significant hurdle, requiring radical tax reforms, perhaps including a "robot tax." Ultimately, the implementation of UBI would necessitate a fundamental cultural shift in how we define value and purpose in a post-labor society.

## Essay C1-8. Deconstructing the myth of meritocracy in higher education.

Educational systems are often lauded as the great equalizers of modern society, operating on the principle that academic success is a direct reflection of individual merit. However, this meritocratic narrative often ignores the systemic inequalities that shape educational outcomes long before a student enters a university lecture hall. Factors such as parental income, access to private tutoring, and the "cultural capital" of one's upbringing provide an invisible head start to children from privileged backgrounds. Consequently, high-stakes standardized testing often measures a student’s socioeconomic stability rather than their raw intellectual potential. This creates a feedback loop where elite institutions inadvertently consolidate class privilege under the guise of objective excellence. To truly democratize education, we must move beyond the rhetoric of "equal opportunity" and address the "equality of conditions." This involves significant investment in early childhood education and a holistic revision of admissions processes to recognize potential in diverse forms. Without such reforms, higher education risks becoming a mechanism for social stratification rather than a vehicle for genuine social mobility and intellectual liberation.

## Essay C1-9. Navigating the "post-truth" era: The decline of objective discourse.

In the contemporary political landscape, the distinction between empirical fact and partisan narrative has become increasingly blurred, leading many to conclude that we have entered a "post-truth" era. This decline in objective discourse is largely fueled by the fragmentation of the media landscape and the algorithmic amplification of polarizing content. When emotional appeal and personal belief carry more weight than evidence, the possibility of a shared reality—essential for democratic deliberation—is undermined. This phenomenon is exacerbated by the "echo chamber" effect, where individuals are insulated from dissenting viewpoints, leading to radicalization and social fragmentation. Furthermore, the deliberate spread of disinformation by state and non-state actors has turned the digital public square into a battlefield of psychological warfare. Reclaiming the value of truth requires more than just better fact-checking; it demands a collective commitment to intellectual humility and the cultivation of a robust skepticism toward our own biases. We must prioritize the strengthening of independent journalistic institutions and the integration of sophisticated information literacy into the core of our educational curricula to ensure that public discourse remains grounded in verifiable reality.

## Essay C1-10. The ethical frontier: Genetic engineering and human enhancement.

The advent of CRISPR technology has propelled the scientific community into an ethical frontier, where the potential to "edit" the human genome is no longer confined to science fiction. While the primary objective of genetic engineering is the eradication of hereditary diseases—a goal that is undeniably noble—it simultaneously opens the door to the controversial realm of human enhancement. The ability to select for specific traits, such as cognitive capacity or physical stamina, raises the specter of "designer babies" and a new form of biological inequality. If genetic enhancements are only available to the wealthy, we risk creating a permanent, genetically superior upper class, thereby institutionalizing a biological caste system. Moreover, the long-term ecological and evolutionary consequences of altering the human germline remain largely unknown. The fundamental question is whether we should interfere with the biological "lottery" that has defined the human condition for millennia. While we should certainly embrace technology that alleviates human suffering, we must establish a global ethical framework to prevent the commodification of the human genome. The power to direct our own evolution is a responsibility that requires profound philosophical reflection and international consensus.

## Essay C1-11. The systemic implications of neoliberalism on social welfare.

The global ascendancy of neoliberal economic policies over the past four decades has fundamentally restructured the social contract between the state and its citizens. Characterized by deregulation, privatization, and significant reductions in public spending, neoliberalism posits that market efficiency is the primary mechanism for social progress. However, the systemic implications for social welfare have been profoundly polarizing. While proponents point to increased global trade and technological innovation as evidence of success, the reality for many is a marked increase in economic precarity. The commodification of essential services, such as healthcare and education, has transformed these fundamental rights into market-based privileges, thereby exacerbating existing inequalities. In many developing nations, the imposition of austerity measures has gutted the social safety nets that once protected the most vulnerable populations. Furthermore, the emphasis on "individual responsibility" often serves to obscure the structural barriers to upward mobility, creating a culture of blame toward the impoverished. Ultimately, the neoliberal paradigm fails to account for the "externalities" of human suffering and environmental degradation. To achieve a more equitable society, we must re-evaluate the role of the state as a guarantor of collective well-being rather than a mere facilitator of market interests.

## Essay C1-12. Analyzing cognitive biases in human decision-making processes.

The traditional economic model of the "rational actor"—an individual who consistently makes logical decisions to maximize utility—has been significantly challenged by findings in behavioral psychology. It is now understood that human decision-making is frequently compromised by a plethora of cognitive biases that operate below the level of conscious awareness. One of the most pervasive is "confirmation bias," the tendency to seek out and prioritize information that aligns with our pre-existing beliefs while disregarding contradictory evidence. In the digital age, this bias is amplified by social media algorithms, leading to increased political polarization. Another significant distortion is the "availability heuristic," where individuals overestimate the probability of events based on how easily they can be recalled from memory, often leading to irrational fears or skewed risk assessments. These biases are not merely individual quirks; they have profound institutional consequences, affecting everything from judicial sentencing to corporate strategy. Recognizing these inherent mental shortcuts is the first step toward more objective reasoning. However, "de-biasing" requires more than just awareness; it necessitates the implementation of structured decision-making frameworks that force the consideration of alternative perspectives and empirical data.

## Essay C1-13. Retribution vs. Rehabilitation: The philosophical divide in criminal justice.

The architecture of contemporary legal systems is often caught in a philosophical tension between the principles of retribution and rehabilitation. The retributive model, rooted in the ancient concept of "lex talionis" or an eye for an eye, posits that the primary purpose of punishment is to impose a penalty proportionate to the crime committed. This approach emphasizes moral accountability and serves a symbolic function of societal vindication. Conversely, the rehabilitative model views criminal behavior as a symptom of underlying social, psychological, or economic pathologies. Its objective is to transform the offender into a productive member of society through education, therapy, and vocational training. Evidence suggests that systems prioritizing rehabilitation, such as those in Scandinavia, exhibit significantly lower recidivism rates compared to more punitive systems. However, the implementation of such programs often faces political resistance from a public that demands "tough on crime" policies. The challenge lies in synthesizing these two approaches: ensuring that justice is served for the victim while acknowledging the humane necessity of offering the offender a path to redemption. Ultimately, a justice system that fails to rehabilitate merely perpetuates a cycle of violence and incarceration.

## Essay C1-14. The paradox of academic pressure and adolescent mental health.

In an increasingly competitive global economy, the pressure on adolescents to achieve academic excellence has reached unprecedented levels. While high standards can motivate students to realize their potential, the current culture of hyper-competitiveness has created a psychological crisis within educational institutions. The relentless focus on standardized testing and elite university admissions has transformed the learning process into a source of chronic stress, anxiety, and burnout. Paradoxically, this intense pressure often undermines the very cognitive functions—such as creativity and critical thinking—that schools aim to cultivate. Furthermore, the "perfectionism" encouraged by these systems is strongly correlated with a rise in adolescent depression and social isolation. When a student's self-worth is tied exclusively to their GPA, any academic setback is perceived as an existential failure. To mitigate this crisis, universities and secondary schools must broaden their definition of success to include emotional intelligence, resilience, and physical well-being. A more holistic approach to education would recognize that a student's mental health is not a secondary concern but the very foundation upon which academic and personal growth are built.

## Essay C1-15. Linguistic relativity: How language shapes our perception of reality.

The Sapir-Whorf hypothesis, or the principle of linguistic relativity, suggests that the structure of a language significantly influences the way its speakers perceive and conceptualize the world. This theory posits that language is not merely a tool for reporting experience but a framework that organizes it. For example, languages that utilize cardinal directions (north, south, east, west) rather than relative terms (left, right) produce speakers with an extraordinary sense of spatial orientation. Similarly, the way different languages categorize colors or time can alter the cognitive processing speeds and memory recall of their speakers. While the "strong" version of this hypothesis—linguistic determinism—is largely rejected by modern linguists, the "weak" version remains a compelling area of study. It suggests that language nudges our attention toward certain features of the environment while allowing others to recede into the background. In a globalized world, understanding these subtle cognitive differences is crucial for effective cross-cultural communication. It reminds us that our "common sense" is often a product of the linguistic heritage we inhabit. By learning a second language, we do not just acquire new labels for the same objects; we gain access to an entirely different way of experiencing the world.

## Essay C1-16. The ontology of the "Digital Self" in social media.

The digital revolution has necessitated a philosophical re-evaluation of identity, specifically through the emergence of the "digital self." Unlike our physical presence, which is bound by biological and temporal constraints, the digital self is a curated, fragmented, and often idealized construction within the virtual realm. This digital persona is not merely a reflection of the individual but an autonomous entity that participates in social discourse and data-driven ecosystems. From an ontological perspective, this creates a dualism between the "actual" self and the "performative" self. The pressure to maintain a consistent and attractive digital narrative can lead to a state of "ontological insecurity," where the individual’s sense of reality becomes dependent on digital validation. Furthermore, the algorithms that govern social platforms play a role in "co-authoring" this identity, as they dictate what content is visible and rewarded. This leads to a feedback loop where the individual modifies their behavior to align with algorithmic expectations. Ultimately, the digital self is a site of constant negotiation between human agency and technological structure. We must ask whether this virtual identity enhances our self-understanding or whether it serves to alienate us from our authentic, embodied existence.

## Essay C1-17. Structural violence in global health policy and resource distribution.

The concept of structural violence, as articulated by Johan Galtung and Paul Farmer, provides a critical framework for understanding the persistent inequalities in global health. Structural violence refers to the systemic ways in which social, political, and economic organizations prevent individuals from achieving their full health potential. This is most evident in the inequitable distribution of medical resources between the Global North and South. The "10/90 gap"—the phenomenon where only 10% of global health research is dedicated to conditions that account for 90% of the world's disease burden—is a direct result of market-driven priorities that favor profitable treatments over essential public health needs. Furthermore, the enforcement of strict intellectual property rights often prevents developing nations from accessing life-saving generic medications. These are not accidental failures but the logical outcomes of a global health architecture that prioritizes capital over human life. Addressing these disparities requires more than just charitable aid; it demands a radical restructuring of international trade laws and a commitment to health as a global public good. To eliminate structural violence in health, we must shift from a model of medical philanthropy to one of distributive justice and systemic equity.

## Essay C1-18. Epistemic injustice in legal testimonies and marginalized voices.

Epistemic injustice occurs when an individual is wronged specifically in their capacity as a knower, often due to systemic prejudices. In the context of the legal system, this frequently manifests as "testimonial injustice," where the credibility of a witness or defendant is unfairly deflated because of their social identity—such as their race, gender, or socio-economic status. For instance, the testimonies of marginalized groups are often met with a "credibility deficit," rooted in deep-seated societal stereotypes that portray these individuals as less reliable or less rational. This not only undermines the fairness of individual trials but also erodes the legitimacy of the judicial institution itself. Furthermore, "hermeneutical injustice" occurs when marginalized individuals lack the conceptual tools to make their experiences intelligible to the dominant legal framework, often because the legal language was constructed without their input. Rectifying these injustices requires a conscious effort by legal professionals to recognize their own implicit biases and to create more inclusive spaces for diverse forms of knowledge. A truly just legal system must ensure that the "epistemic agency" of all citizens is respected, regardless of their position in the social hierarchy.

## Essay C1-19. The aesthetic of the "Uncanny Valley" in AI and robotics.

The "Uncanny Valley," a hypothesis introduced by Masahiro Mori, describes the psychological discomfort experienced by humans when encountering robots or AI avatars that appear nearly, but not perfectly, human. As technological representations of the human form become increasingly sophisticated, they reach a point where subtle imperfections—such as unnatural eye movements or a lack of emotional micro-expressions—evoke a sense of revulsion or "eeriness." This phenomenon reveals profound insights into human psychology and our evolutionary need to distinguish between the "living" and the "simulated." From an aesthetic standpoint, the uncanny valley challenges our definitions of beauty and realism, suggesting that human likeness is not a linear progression but a delicate balance. In the realm of AI-driven entertainment and healthcare, navigating this valley is a significant hurdle. If an AI therapist or a digital actor feels "uncanny," it can hinder trust and emotional connection. However, some artists deliberately exploit the uncanny to provoke reflection on the nature of consciousness and the boundary between human and machine. As we move toward a future populated by humanoid entities, understanding the mechanics of the uncanny will be essential for designing technologies that harmonize with, rather than disrupt, our psychological well-being.

## Essay C1-20. Theoretical frameworks of "Soft Power" in international relations.

The concept of "soft power," popularized by Joseph Nye, describes the ability of a nation to influence international actors through attraction and persuasion rather than military or economic coercion (hard power). Soft power is derived from three primary sources: a country's culture, its political values, and its foreign policies. In the 21st century, the importance of soft power has surged as the globalized information environment makes traditional warfare increasingly costly and unpopular. Nations invest heavily in "public diplomacy," utilizing media, educational exchanges, and cultural exports—such as cinema and music—to build a favorable international image. For example, the global reach of American pop culture or the "Hallyu" wave from South Korea are potent examples of cultural soft power. However, the efficacy of soft power is often difficult to quantify, as attraction is subjective and slow to yield tangible results. Critics also argue that soft power can be a form of "cultural imperialism," where dominant nations impose their values on others under the guise of attraction. For a state to truly wield soft power, its domestic actions must align with its international rhetoric; hypocrisy is the greatest deterrent to soft power. Ultimately, the most successful states are those that can effectively integrate both hard and soft power into a comprehensive "smart power" strategy.

## Essay C1-21. The psychological impact of algorithmic filter bubbles.

The architectural design of modern social media platforms is increasingly predicated on algorithms that prioritize user engagement through the personalization of content. While this enhances user experience by providing relevant information, it simultaneously creates "filter bubbles" that significantly distort our perception of social reality. These digital enclaves isolate individuals from dissenting viewpoints, reinforcing existing cognitive biases and fostering an environment of radicalization. Psychologically, the constant validation provided by these echo chambers can lead to a state of "intellectual narcissism," where the individual becomes increasingly incapable of engaging in nuanced, cross-perspective dialogue. This fragmentation of the public square poses a fundamental threat to democratic discourse, which relies on a shared set of facts and the willingness to compromise. Furthermore, the lack of exposure to "cognitive friction"—the encounter with challenging or uncomfortable ideas—stunts intellectual growth and critical thinking. To mitigate these effects, it is imperative that both developers and users prioritize "algorithmic transparency" and actively seek out diverse information sources. Ultimately, the health of our digital society depends on our ability to burst these bubbles and reclaim a more pluralistic and objective information landscape.

## Essay C1-22. Exploring the "tragedy of the commons" in environmental policy.

The "tragedy of the commons"—a concept popularized by Garrett Hardin—describes a situation where individual actors, acting rationally according to their own self-interest, deplete a shared resource, ultimately harming the entire collective. This theoretical framework is profoundly relevant to contemporary environmental challenges, such as overfishing, deforestation, and the pollution of the atmosphere. In a globalized economy, the lack of a centralized authority to regulate "common-pool resources" often leads to a "race to the bottom," where nations or corporations prioritize short-term profit over long-term ecological stability. For instance, the carbon emissions of a single country may provide domestic economic growth while contributing to a global climate crisis that affects everyone. Addressing this tragedy requires a shift from voluntary agreements to enforceable international frameworks that internalize the "externalities" of environmental degradation. This includes the implementation of carbon taxes, cap-and-trade systems, and the establishment of marine protected areas. However, the primary obstacle remains the tension between national sovereignty and global responsibility. To ensure a sustainable future, we must move toward a model of "earth system governance" that recognizes the fundamental interdependence of all biological and economic systems.

## Essay C1-23. The role of neuroplasticity in lifelong learning and cognitive resilience.

The traditional view that the human brain becomes a static organ after early adulthood has been definitively overturned by the discovery of neuroplasticity—the brain's remarkable ability to reorganize itself by forming new neural connections throughout life. This paradigm shift has profound implications for our understanding of lifelong learning and the prevention of age-related cognitive decline. Neuroplasticity suggests that the brain remains a highly dynamic system that responds to environmental stimuli, intellectual challenges, and physical exercise. Engaging in cognitively demanding tasks, such as learning a new language or mastering a complex musical instrument, stimulates the growth of new synapses and enhances "cognitive reserve." This reserve acts as a buffer against neurological damage, potentially delaying the onset of symptoms associated with dementia and Alzheimer’s. Furthermore, neuroplasticity provides a biological basis for the efficacy of rehabilitation after brain injuries. However, the "use it or lose it" principle remains a critical caveat; a lack of mental stimulation can lead to the pruning of neural pathways and a decline in cognitive flexibility. Therefore, fostering a culture of continuous education is not merely a social ideal but a physiological necessity for maintaining a healthy and resilient aging population.

## Essay C1-24. Evaluating the efficacy of carbon taxes on corporate behavior.

As the urgency of the climate crisis intensifies, the implementation of carbon taxes has emerged as a primary economic instrument for incentivizing the transition to a low-carbon economy. The underlying logic of a carbon tax is to place a financial burden on the negative "externality" of greenhouse gas emissions, thereby making fossil fuel consumption less attractive than renewable alternatives. Proponents argue that this market-based approach encourages corporate innovation, as companies seek to reduce their tax liability by investing in green technologies and energy efficiency. Furthermore, the revenue generated from these taxes can be "revenue-neutral," meaning it is returned to citizens or reinvested in public transit and environmental restoration. However, critics highlight the risk of "carbon leakage," where corporations relocate their production to countries with more lenient environmental regulations, thereby negating the global benefit. There is also the concern that carbon taxes can be regressive, disproportionately affecting low-income households through increased energy costs. To be effective, a carbon tax must be part of a comprehensive policy package that includes "border carbon adjustments" and targeted subsidies for vulnerable populations. Ultimately, the success of such a tax depends on its ability to catalyze a systemic shift in the global energy landscape.

## Essay C1-25. The intersection of ethics and biometric surveillance in the public square.

The rapid deployment of biometric surveillance technologies, such as facial recognition and iris scanning, in public spaces has sparked an intense ethical debate regarding the balance between national security and individual liberty. Proponents of these systems highlight their efficacy in identifying criminal suspects and preventing terrorist activities, arguing that the sacrifice of a degree of anonymity is a small price to pay for public safety. However, the pervasive nature of biometric tracking creates a "surveillance state" where the expectation of privacy in public is effectively eliminated. This can have a "chilling effect" on the freedoms of assembly and expression, as individuals may self-censor their behavior to avoid becoming a "person of interest" in a government database. Moreover, the technical limitations of these systems, including higher error rates for certain demographic groups, raise profound concerns about algorithmic bias and systemic discrimination. The permanent and immutable nature of biometric data also poses a unique security risk; unlike a password, one’s face or fingerprints cannot be changed in the event of a data breach. To protect the democratic fabric of society, it is essential to establish rigorous legal frameworks that limit the use of biometric surveillance to high-stakes scenarios and ensure strict judicial oversight.

## Essay C1-26. The paradox of choice in contemporary consumer psychology.

In modern affluent societies, the abundance of choice is often equated with individual freedom and market efficiency. However, psychological research into the "paradox of choice" suggests that an excessive array of options can, in fact, lead to increased anxiety and decision paralysis. When faced with hundreds of nearly identical products, consumers often experience a heightened fear of making a sub-optimal choice, leading to "post-purchase regret." This phenomenon is amplified by digital platforms that provide endless comparisons and reviews, creating a state of perpetual dissatisfaction. From an economic perspective, while variety is intended to maximize utility, the cognitive load required to process these choices often diminishes the overall well-being of the individual. Furthermore, the constant pressure to choose "the best" fosters a culture of perfectionism that is ultimately unsustainable. To mitigate this, many individuals are gravitating toward "minimalism" and curated experiences that limit choice to enhance mental clarity. Ultimately, we must recognize that true autonomy is not found in the quantity of options available, but in the ability to make meaningful decisions aligned with one's core values.

## Essay C1-27. Deconstructing the "Gig Economy" and the erosion of labor rights.

The rise of the "gig economy," facilitated by digital platforms such as Uber and Upwork, has fundamentally altered the traditional employment landscape. While proponents celebrate the flexibility and autonomy it offers to workers, a more critical analysis reveals the systematic erosion of hard-won labor rights. Gig workers are often classified as "independent contractors" rather than employees, a legal distinction that allows corporations to circumvent responsibilities such as health insurance, paid leave, and minimum wage protections. This shift effectively transfers the economic risk from the corporation to the individual, creating a new class of "precariat" workers who lack long-term financial security. Furthermore, the algorithmic management of labor—where work is assigned and evaluated by unfeeling code—precludes the possibility of human negotiation and collective bargaining. While the gig economy provides a vital income source for many, it simultaneously threatens to dismantle the social safety nets that define a just society. Addressing this requires a radical update to labor laws that recognize the nuances of platform-based work. We must ensure that technological innovation does not become a pretext for the exploitation of the modern workforce.

## Essay C1-28. The role of irony and satire in contemporary political discourse.

In an era characterized by extreme political polarization and the decline of traditional journalism, irony and satire have emerged as potent tools for social critique. Satirical news programs and digital memes often possess a greater capacity to expose the absurdities of power than conventional reporting, as they can bypass the filters of political correctness and "both-sides" neutrality. By utilizing humor to highlight hypocrisy, satire can foster a more critical and engaged citizenry. However, the pervasive use of irony also poses a significant risk to the health of public discourse. The "ironic stance" can become a form of cynical detachment, where nothing is taken seriously and all truth-claims are met with mockery. This can lead to a state of "post-earnestness," where genuine political commitment is seen as uncool or naive. Furthermore, in the hands of malicious actors, irony can be used to mask extremist ideologies, allowing them to retreat into the defense of "it was just a joke" when challenged. Ultimately, while satire is essential for a vibrant democracy, it must be balanced with a capacity for sincere deliberation and a commitment to shared moral principles.

## Essay C1-29. Bio-ethics in the age of neural implants and cognitive enhancement.

The development of Brain-Computer Interfaces (BCIs) and neural implants represents one of the most profound technological leaps in human history. While the initial applications are focused on restoring function to individuals with neurological disorders, the potential for cognitive enhancement in "healthy" individuals raises unprecedented bio-ethical dilemmas. The ability to directly augment memory, processing speed, or even emotional regulation could redefine the human experience. However, this technology threatens to create a "neuro-cognitive divide," where those who can afford enhancements possess a permanent biological advantage over those who cannot. This would institutionalize inequality at the level of the human brain, potentially leading to a new form of social stratification. Furthermore, the integration of neural implants poses significant risks to cognitive liberty and privacy; if our thoughts can be interfaced with a computer, they can also be monitored or manipulated by external actors. The prospect of "hacking" the human mind is no longer a distant theoretical concern but a looming reality. As we venture into this neuro-technological future, we must establish a global ethical framework that prioritizes human dignity, consent, and equitable access.

## Essay C1-30. The shifting paradigm of sovereignty in a globalized digital world.

The traditional Westphalian concept of sovereignty, defined by a state's absolute authority over a bounded physical territory, is increasingly becoming an anachronism in the digital age. The rise of transnational digital corporations, global financial flows, and decentralized technologies like blockchain has created a "borderless" reality that challenges the regulatory power of the nation-state. Today, data is often more valuable than land, and the entities that control the flow of information—rather than those that control physical borders—wield significant geopolitical influence. This has led to the emergence of "digital sovereignty," where states attempt to reclaim control over their citizens' data through localized cloud infrastructures and national firewalls. However, these efforts are often in tension with the inherently global nature of the internet. Furthermore, the rise of non-state actors, such as "hacktivist" groups and private military contractors, further complicates the state's monopoly on the use of force and information. We are witnessing a transition toward a more complex, multi-layered model of sovereignty, where power is negotiated between states, corporations, and global civil society. The challenge for the 21st century is to develop new forms of international governance that can maintain order and protect rights in this increasingly fluid and virtual landscape.

## Essay C1-31. The crisis of modern leadership: Charisma vs. Competence.

In contemporary political and corporate spheres, there is an observable tension between the demand for charismatic leadership and the necessity for technical competence. Historically, charismatic leaders have been valued for their ability to inspire collective action and articulate a compelling vision for the future. In times of crisis, the emotional resonance provided by a charismatic figure can be a potent force for social mobilization. However, the "cult of personality" often prioritizes style over substance, leading to the rise of leaders who possess rhetorical flair but lack the complex problem-solving skills required for effective governance. Conversely, a purely technocratic leader, while highly competent, may struggle to build the public trust and emotional connection necessary to implement difficult reforms. The most successful leadership models are those that synthesize these two attributes—utilizing charisma to build consensus while grounding policy in empirical evidence and expert knowledge. In an era of "post-truth" politics, the need for competent leadership that can navigate the nuances of global challenges has never been more urgent. Ultimately, the measure of a true leader is not their ability to win an audience, but their capacity to deliver sustainable and ethical results.

## Essay C1-32. Assessing the socio-economic impacts of the "Sharing Economy."

The emergence of the "sharing economy," characterized by peer-to-peer platforms such as Airbnb and BlaBlaCar, was initially hailed as a revolutionary shift toward a more sustainable and community-oriented economic model. By allowing individuals to monetize underutilized assets, these platforms promised to reduce waste and provide supplementary income for the middle class. However, the socio-economic reality has proven to be more complex. In many urban centers, the proliferation of short-term rentals has led to a significant increase in housing prices and the "touristification" of local neighborhoods, often displacing long-term residents. Furthermore, the lack of traditional regulatory oversight has raised concerns regarding safety standards and tax evasion. From a labor perspective, the sharing economy often operates on the fringes of employment law, providing flexibility at the cost of job security and benefits. While these platforms have undoubtedly enhanced consumer choice and lowered transaction costs, they have also contributed to the commodification of social interactions and the erosion of local community structures. To maximize the benefits of this model, governments must implement nuanced regulatory frameworks that protect public interests without stifling technological innovation.

## Essay C1-33. The role of cognitive dissonance in political polarization.

Cognitive dissonance—the psychological discomfort experienced when holding two or more contradictory beliefs—plays a fundamental role in the deepening of political polarization. When individuals are confronted with evidence that challenges their ideological worldview, they often engage in "motivated reasoning" to reduce this discomfort. Rather than updating their beliefs in light of new facts, they tend to double down on their existing convictions, seeking out information that confirms their biases while delegitimizing contradictory sources. This mechanism is significantly amplified by the "echo chamber" effect of digital media, where individuals are rarely exposed to the cognitive friction of dissenting opinions. Consequently, political discourse becomes less about the rational evaluation of policy and more about the defense of tribal identity. This leads to a state of "epistemic closure," where compromise becomes impossible and the "other side" is viewed not as a political opponent but as an existential threat. Addressing polarization requires a conscious effort to cultivate "intellectual humility"—the recognition that our own perceptions are fallible. Without a collective willingness to sit with the discomfort of dissonance, the possibility of a shared objective reality continues to recede.

## Essay C1-34. The ethical landscape of "Nudge Theory" in public policy.

"Nudge theory," popularized by Richard Thaler and Cass Sunstein, suggests that positive reinforcement and indirect suggestions can influence the behavior and decision-making of groups or individuals as effectively, if not more so, than direct instruction or legislation. Governments worldwide have established "nudge units" to encourage behaviors such as organ donation, tax compliance, and healthy eating by altering the "choice architecture" of society. While the efficacy of these interventions is well-documented, they raise profound ethical questions regarding paternalism and autonomy. Critics argue that "nudging" is a form of psychological manipulation that bypasses conscious deliberation, thereby undermining the individual's capacity for agency. Even if the nudge is for the "greater good," the lack of transparency in how these choices are designed can lead to a "democratic deficit." Furthermore, there is a risk that governments may use nudges as a low-cost substitute for necessary structural reforms. For nudge theory to be ethically defensible, it must be implemented with a high degree of transparency and public consent. The goal should be to empower citizens to make better choices for themselves, rather than covertly engineering their behavior to suit state objectives.

## Essay C1-35. Decolonizing the curriculum: Knowledge, power, and representation.

The movement to "decolonize the curriculum" in higher education is not merely an attempt to add diverse voices to a reading list; it is a fundamental challenge to the Eurocentric hegemony of knowledge production. For centuries, Western academic institutions have operated under the assumption that European perspectives represent a universal objective truth, while marginalizing the intellectual traditions of the Global South. This epistemic exclusion is a legacy of colonial power structures that sought to delegitimize the knowledge systems of the colonized. Decolonizing the curriculum involves a critical interrogation of the "canon" and the recognition that all knowledge is situated within specific historical and political contexts. By integrating indigenous philosophies, non-Western histories, and post-colonial theories, universities can foster a more pluralistic and inclusive intellectual environment. However, this process often faces resistance from those who view it as a threat to academic standards or a form of political indoctrination. On the contrary, a decolonized curriculum enhances academic rigor by encouraging students to think critically about the relationship between power and knowledge. Ultimately, the goal is to move toward a truly globalized university that reflects the diverse intellectual heritage of all of humanity.

## Essay C1-36. The "Attention Economy" and the erosion of cognitive autonomy.

In the contemporary digital landscape, the primary commodity is no longer information itself, but the human attention required to process it. This "attention economy" is driven by sophisticated algorithms designed specifically to capture and monetize our focus through the constant delivery of dopamine-triggering notifications and personalized content. While proponents argue that these platforms provide unparalleled connectivity and entertainment, a more critical analysis suggests that they are fundamentally eroding our cognitive autonomy. The design of "infinite scroll" and "auto-play" features exploits our psychological vulnerabilities, leading to a state of perpetual distraction and a significant reduction in our capacity for "deep work" or sustained contemplation. From a neuro-philosophical perspective, this constant fragmentation of attention disrupts our ability to form a coherent narrative of the self, as our mental lives are increasingly dictated by external technological stimuli rather than internal volition. To reclaim our autonomy, we must implement "digital minimalism" and demand the design of technologies that respect human agency. Ultimately, our attention is our most precious resource; if we do not consciously direct it, it will be harvested and sold by the entities that control the digital architecture.

## Essay C1-37. Ethical implications of "Gene Drives" in biodiversity conservation.

The development of "gene drive" technology—a method of genetic engineering that ensures a specific trait is passed down to all offspring—has opened a new and controversial frontier in biodiversity conservation. Proponents suggest that gene drives could be used to eradicate invasive species or eliminate disease-carrying insects, such as the mosquitoes that transmit malaria. The potential to save thousands of human lives and protect fragile ecosystems is an undeniably powerful argument. However, the ethical and ecological risks are profound. Releasing a gene drive into the wild is an essentially irreversible act with unpredictable consequences for the entire food web. If we eliminate one species, we may inadvertently cause the collapse of others that depend on it. Furthermore, the technology raises questions of "biological sovereignty": who has the right to decide to alter the genetic makeup of a species that exists across national borders? There is also the risk of the technology being repurposed for harmful ends. To navigate this ethical minefield, we must establish a rigorous international regulatory framework that prioritizes the "precautionary principle" and ensures that the voices of all stakeholders, particularly local communities, are heard.

## Essay C1-38. Post-colonial perspectives on international law and human rights.

The modern framework of international law and universal human rights is often presented as a neutral, objective set of principles designed to protect human dignity globally. However, post-colonial scholars argue that these frameworks are deeply embedded in Western philosophical traditions and continue to reflect the power dynamics of the colonial era. From this perspective, the "universality" of human rights often serves as a form of "normative imperialism," where Western values are imposed on diverse cultures under the guise of progress. For instance, the emphasis on individual civil liberties often takes precedence over collective socio-economic rights, which are frequently a priority for the Global South. Furthermore, the enforcement of international law is often selective, with powerful Western nations frequently exempting themselves from the very standards they impose on others. To achieve a truly global justice system, we must "decolonize" international law by integrating legal traditions and ethical perspectives from Africa, Asia, and Latin America. This requires a shift from a top-down imposition of norms to a genuine cross-cultural dialogue that recognizes the pluralistic nature of human dignity.

## Essay C1-39. The aesthetic of brutality and its revival in modern architecture.

Brutalist architecture, characterized by its raw concrete surfaces, massive forms, and visible structural elements, has long been one of the most polarizing aesthetic movements in urban design. After decades of being dismissed as "ugly" or "oppressive," Brutalism is currently experiencing a significant revival in both academic discourse and popular culture. This resurgence can be attributed to a growing appreciation for the movement's "material honesty" and its historical association with the social-democratic ideals of the mid-20th century. Unlike the sleek, glass-clad towers of contemporary neoliberal architecture, Brutalist buildings possess a sense of permanence and civic weight. However, the aesthetic remains controversial due to its perceived lack of human scale and its tendency to weather poorly in certain climates, leading to a sense of urban decay. From a psychological standpoint, the "sublime" scale of Brutalist structures can evoke a sense of awe, but it can also feel alienating to the individual. The challenge for modern architects is to learn from the functionalist strengths of Brutalism while integrating more sustainable materials and "biophilic" elements that enhance human well-being. Ultimately, the revival of Brutalism represents a desire for an architecture of substance in an increasingly ephemeral and digital world.

## Essay C1-40. Technocratic governance vs. Democratic accountability in the 21st century.

As the challenges facing modern states—such as climate change, global pandemics, and financial stability—become increasingly technical, there is a growing trend toward "technocratic governance," where decision-making power is shifted from elected representatives to unelected experts. The argument for technocracy is rooted in efficiency; experts possess the specialized knowledge required to navigate complex systems without the distractions of partisan politics or short-term electoral cycles. However, this shift poses a significant threat to democratic accountability. When crucial policy decisions are made in "expert committees" or central banks, the public's ability to influence the direction of their society is diminished, leading to a sense of political alienation. This "democratic deficit" can fuel the rise of populist movements that reject expert advice altogether, even when it is scientifically sound. The central challenge of 21st-century politics is to find a synthesis that respects both scientific expertise and democratic legitimacy. This requires the development of "deliberative" institutions where experts and citizens can engage in transparent dialogue. We must ensure that while experts "inform" policy, the ultimate moral and political choices remain the prerogative of the sovereign people.

## Essay C1-41. Phenomenological aspects of Virtual Reality in human experience.

The advent of Virtual Reality (VR) technology has introduced a novel dimension to human experience, necessitating a phenomenological exploration of "presence" and "immersion." Unlike traditional media, which maintains a clear boundary between the observer and the observed, VR attempts to dissolve this barrier, placing the individual within a digitally synthesized environment. From a phenomenological perspective, this challenges our fundamental understanding of spatiality and embodiment. When a user experiences "presence" in a virtual world, their consciousness begins to accept the digital stimuli as a primary reality, leading to an interesting "split" between the physical body and the virtual avatar. This raises profound questions about the nature of perception: if our brains can be fully convinced by a simulation, what does this imply about our "objective" experience of the physical world? Furthermore, the potential for VR to facilitate "radical empathy"—by allowing users to inhabit the life-experience of others—could have significant social implications. However, there are concerns regarding "ontological confusion" and the potential for psychological escapism. Ultimately, VR is not merely a technological advancement but a new frontier in the human quest to understand the relationship between consciousness and the external world.

## Essay C1-42. Assessing the 'Resource Curse' in developing economies.

The 'Resource Curse,' or the paradox of plenty, refers to the phenomenon where countries with an abundance of natural resources—such as oil, minerals, or gas—tend to experience lower economic growth and poorer governance than countries with fewer resources. This systemic issue is often rooted in "Dutch Disease," where a surge in resource exports leads to a currency appreciation that makes other sectors, like manufacturing and agriculture, less competitive. Furthermore, the reliance on resource rents often fosters a "rentier state" model, where the government is not dependent on taxation and therefore feels less accountable to its citizens. This environment is highly conducive to corruption, patronage, and the neglect of human capital investment. In many developing nations, the struggle for control over these resources can even lead to civil conflict and political instability. Breaking the resource curse requires a radical commitment to institutional transparency and the diversification of the economy. Successful models, such as the Norwegian Sovereign Wealth Fund, demonstrate that with robust democratic oversight, natural wealth can be a blessing rather than a curse. Ultimately, the challenge is to transform finite natural resources into sustainable human development.

## Essay C1-43. The ethics of algorithmic transparency in social credit systems.

The implementation of social credit systems, which utilize big data and algorithms to evaluate the "trustworthiness" and social behavior of citizens, represents a significant shift in the nature of governance and social control. While proponents argue that such systems can enhance social order and incentivize civic responsibility, the ethical implications for privacy and autonomy are profound. The central concern lies in the "black box" nature of the algorithms used to calculate these scores. Without transparency regarding the criteria for "good" or "bad" behavior, citizens are subjected to a form of automated judgment that they cannot effectively challenge. This lack of algorithmic transparency creates an environment of self-censorship and conformity, as individuals fear that their private actions or dissenting opinions will negatively impact their social standing or access to essential services. Moreover, the potential for these systems to be used for political repression is a major threat to democratic values. To protect human rights in the age of big data, any system of social evaluation must be grounded in legal oversight, radical transparency, and the right to appeal. We must ensure that technology serves to empower individuals rather than becoming a tool for pervasive and opaque social engineering.

## Essay C1-44. Re-evaluating the 'Social Contract' in a post-labor society.

As Artificial Intelligence and robotics continue to automate traditional labor markets, we are facing the prospect of a "post-labor" society where human work is no longer the primary driver of economic production. This potential shift necessitates a fundamental re-evaluation of the 'Social Contract'—the implicit agreement between the individual and the state regarding rights and responsibilities. Historically, the social contract has been predicated on the idea that the individual provides labor in exchange for economic security and social status. In a world with diminished labor demand, this foundational link is severed, raising the risk of mass disenfranchisement and extreme inequality. To maintain social cohesion, we must decouple survival from employment, perhaps through the implementation of a Universal Basic Income or a "social wealth fund." Furthermore, we must redefine what it means to be a "contributing member" of society, placing a higher value on creative, educational, and caregiving roles that are currently marginalized by the market. The transition to a post-labor society is not merely a technical challenge but a philosophical one; it requires us to construct a new moral framework for a world where our identity and purpose are no longer defined by our profession.

## Essay C1-45. Intellectual property rights and global vaccine equity.

The global response to recent pandemics has brought the tension between intellectual property (IP) rights and public health into sharp focus. While IP protections are designed to incentivize innovation by allowing pharmaceutical companies to recoup their research and development costs, the enforcement of these rights during a global health crisis can create significant barriers to vaccine equity. In many developing nations, the inability to produce or afford patented vaccines has led to prolonged suffering and the emergence of new viral variants, which ultimately threatens global security. Critics argue that when life-saving technologies are funded by public research, they should be treated as global public goods rather than private commodities. The proposal for a "TRIPS waiver"—which would temporarily suspend patent protections for medical products—has been championed as a necessary step to decentralize production and ensure that no one is left behind. However, pharmaceutical corporations maintain that such a move would undermine the incentives for future innovation. Resolving this conflict requires a new model of "cooperative innovation," where IP is shared through patent pools and technology transfer initiatives. Ultimately, in a globalized world, the health of the individual is inextricably linked to the health of the collective, and our legal frameworks must reflect this reality.

## Essay C1-46. Cultivating epistemic humility in an era of ideological certainty.

The current socio-political landscape is increasingly characterized by a profound lack of "epistemic humility"—the intellectual recognition that one’s knowledge is inherently partial and fallible. In an era where digital algorithms curate our information environment to align with our pre-existing beliefs, the capacity for self-correction is being systematically undermined. When we are insulated from the "cognitive friction" of dissenting perspectives, we tend toward a state of ideological certainty that views complexity as a threat rather than a feature of reality. This dogmatism not only stifles intellectual growth but also fuels the polarization that paralyzes democratic deliberation. Cultivating epistemic humility requires a conscious effort to engage with the strongest versions of opposing arguments and to acknowledge the structural biases that shape our own perceptions. It is not a sign of weakness, but a prerequisite for genuine learning and social cohesion. By embracing the possibility that we may be wrong, we open the door to a more nuanced and compassionate understanding of the human condition. Ultimately, the health of our public discourse depends on our willingness to prioritize the pursuit of truth over the performance of certainty.

## Essay C1-47. Biocentrism vs. Anthropocentrism: A philosophical shift for the Anthropocene.

The environmental crisis of the 21st century has prompted a radical re-evaluation of the relationship between humanity and the natural world, specifically the tension between anthropocentric and biocentric worldviews. Anthropocentrism—the belief that humans are the center of the moral universe and that nature possesses value only in its utility to us—has provided the philosophical justification for centuries of ecological exploitation. This perspective views the environment as a "standing reserve" of resources to be managed for economic growth. Conversely, biocentrism posits that all living beings have inherent value, independent of their usefulness to humans. This shift toward a more "earth-centered" ethics is essential if we are to survive the Anthropocene. A biocentric approach necessitates a fundamental restructuring of our legal and economic systems, recognizing the rights of nature and the interdependence of all biological life. While critics argue that biocentrism is impractical in a globalized economy, the alternative—a continued adherence to anthropocentric exploitation—is an existential dead end. Transitioning toward a biocentric paradigm is not merely an ethical choice, but a practical necessity for the long-term habitability of the planet.

## Essay C1-48. The "Panopticon" and the psychology of digital self-censorship.

Jeremy Bentham’s "Panopticon"—a prison design where inmates are aware they might be watched at any moment but can never be sure—serves as a potent metaphor for the contemporary digital surveillance state. In the age of big data, the pervasive tracking of our digital movements has created a "virtual panopticon" where the mere awareness of surveillance alters our behavior. This leads to a profound psychological phenomenon: digital self-censorship. When individuals believe that their private communications, search histories, and social interactions are being monitored by state or corporate actors, they tend to avoid controversial topics, stifle dissent, and adhere to "safe" social norms. This chilling effect on the freedom of expression occurs without the need for overt coercion; the "gaze" of the algorithm is sufficient to enforce conformity. From a sociological perspective, this erosion of the "private self" undermines the very foundation of autonomous agency and democratic participation. To reclaim our cognitive liberty, we must establish robust legal protections for digital privacy and challenge the normalization of pervasive monitoring. In the absence of such protections, the digital public square risks becoming a space of performative compliance rather than authentic deliberation.

## Essay C1-49. Neuroethics and the implications of memory manipulation.

Recent advancements in neuroscience, specifically in the fields of optogenetics and neuropharmacology, have brought the possibility of memory manipulation into the realm of clinical reality. While the potential to alleviate the suffering of individuals with Post-Traumatic Stress Disorder (PTSD) by "erasing" or dampening traumatic memories is undeniably noble, the neuroethical implications are staggering. Memory is not merely a record of the past; it is the core of our personal identity and a vital component of our moral development. If we can selectively edit our experiences, we risk undermining the authenticity of the self and the lessons learned from adversity. Furthermore, the possibility of "enhancing" memories for competitive advantage raises profound questions about equity and the commodification of human consciousness. There is also the significant risk of misuse by state actors for the purpose of psychological indoctrination or the erasure of historical atrocities. As we venture further into this neuro-technological frontier, we must establish rigorous ethical frameworks that prioritize "narrative integrity" and the right to psychological continuity. The power to edit our past is a responsibility that requires profound philosophical reflection, as it touches upon the very essence of what it means to be human.

## Essay C1-50. The emergence of post-national identity in a globalized digital society.

The traditional concept of identity, historically rooted in national, ethnic, and geographic boundaries, is undergoing a profound transformation in the digital age. We are witnessing the emergence of "post-national" identities, where individuals define themselves through global networks, shared digital cultures, and transnational social movements. For the "digital native" generation, the internet provides a space for self-construction that is increasingly independent of the nation-state’s narrative. This shift has been facilitated by the decline of traditional media and the rise of decentralized communication platforms that allow for the formation of "imagined communities" across borders. While some view this as a positive step toward a more cosmopolitan and inclusive world, others perceive it as a threat to local cultural diversity and social cohesion. This tension is often manifested in the rise of reactionary nationalist movements that seek to reclaim a sense of traditional belonging. However, the fluid and hybridized nature of digital identity suggests that the return to a mono-cultural national identity is an impossibility. The challenge for the 21st century is to develop new forms of political and social organization that can accommodate these multi-layered, post-national identities without succumbing to fragmentation.
